# Identification of residues involved in allosteric signal transmission from amino acid binding site of pyruvate kinase muscle isoform 2

**DOI:** 10.1371/journal.pone.0282508

**Published:** 2023-03-10

**Authors:** Suparno Nandi, Mishtu Dey

**Affiliations:** Department of Chemistry, The University of Iowa, Iowa City, IA, United States of America; Drexel University, UNITED STATES

## Abstract

PKM2 is a rate-limiting enzyme in the glycolytic process and is involved in regulating tumor proliferation. Several amino acids (AAs) such as Asn, Asp, Val, and Cys have been shown to bind to the AA binding pocket of PKM2 and modulate its oligomeric state, substrate binding affinity, and activity. Although previous studies have attributed that the main chain and side chain of bound AAs are responsible for initiating signal to regulate PKM2, the signal transduction pathway remains elusive. To identify the residues involved in signal transfer process, N70 and N75 located at two ends of a β strand connecting the active site and AA binding pocket were altered. Biochemical studies of these variants with various AA ligands (Asn, Asp, Val, and Cys), illustrate that N70 and N75, along with β1 connecting these residues are part of the signal transduction pathway between the AA binding pocket and the active site. The results demonstrate that mutation of N70 to D prevents the transfer of the inhibitory signal mediated by Val and Cys, whereas N75 to L alteration blocks the activating signal initiated by Asn and Asp. Taken together, this study confirms that N70 is one of the residues responsible for transmitting the inhibitory signal and N75 is involved in the activation signal flow.

## Introduction

Pyruvate kinase muscle isoform 2 (PKM2) is an essential glycolytic enzyme involved in cancer metabolism. It catalyzes the rate-limiting step of glycolysis by converting phosphoenolpyruvate (PEP) and adenosine diphosphate (ADP) to adenosine triphosphate (ATP) [1, 2]. PKM2 is predominantly expressed in normal proliferating, embryonic, and cancer cells [3]. Tumors utilize PKM2 to fuel their growth and proliferation, and at a molecular level this function depends on the unique property of the enzyme to exist in an active tetramer and an inactive dimer/monomer form [4].

The activity and oligomeric state of PKM2 are modulated by several metabolites (such as fructose-1, 6- bisphosphate (FBP) [5], succinyl-5- aminoimidazole-4-carboxamide-1-ribose 5’- phosphate (SAICAR) [6, 7]), post-translational modifications (acetylation [8], phosphorylation [9–11], hydroxylation [12], etc.), synthetic small molecules (such as DASA-58, TEPP-46 [13], 1- (sulfonyl)-5-(arylsulfonyl)indoline [14]), and amino acids (AAs) (cysteine [15], serine [16], valine, asparagine, aspartate [17]). Pyruvate, produced by active tetrameric PKM2 [2], is converted to lactate, which drives the local invasion of cancer cells [18]. On the other hand, the less active dimeric form of PKM2 translocate into the nucleus, where it acts as a transcriptional coactivator, consequently upregulating the transcription of proto-oncogenes [8, 10, 19]. Moreover, the presence of dimeric/monomeric PKM2 leads to a buildup of glycolytic intermediates that act as raw materials for the synthesis of nucleotides, lipids, and amino acids necessary for cell division [2]. Thus, the survival of cancer cells depends on a finely tuned regulation mechanism of PKM2.

In each PKM2 monomer, there are four domains, namely, N-(residues 1–44), A-(residues 44–116 and 219–389), B-(residues 117–218), and C-(residues 390–531) domains. The active site and FBP binding sites are located in A- and C-domains, respectively (S1A and S1B Fig). The AA binding pocket is located between the A- and C-domains at a distance of 24 Å from the active site [5, 15]. Serine/asparagine/aspartate [16, 17] and FBP [5] have been shown to promote the active tetrameric form of PKM2 by binding to the AA- and FBP- binding pockets of PKM2, respectively. In contrast, acetylation [20] and phosphorylation [20] of PKM2 result in a disruption of the tetrameric state caused by the removal of FBP from its binding pocket due to the movement of specific FBP binding residues [20]. Likewise, cysteine and valine inhibit PKM2 by converting it to a mixture of tetramer:dimer/monomer form [15, 17, 21]. In a recent report we have demonstrated that differences in the sidechain polarity of AAs result in a distinct allosteric response in PKM2 [17]. While we have shown that a bidirectional coupling between the AA binding site and the active site exists, promoting either activation/inhibition of PKM2 in the presence of different amino acids, the residues involved in transferring the allosteric signal from the AA binding site to the active site remain undetermined.

**Fig 1:**
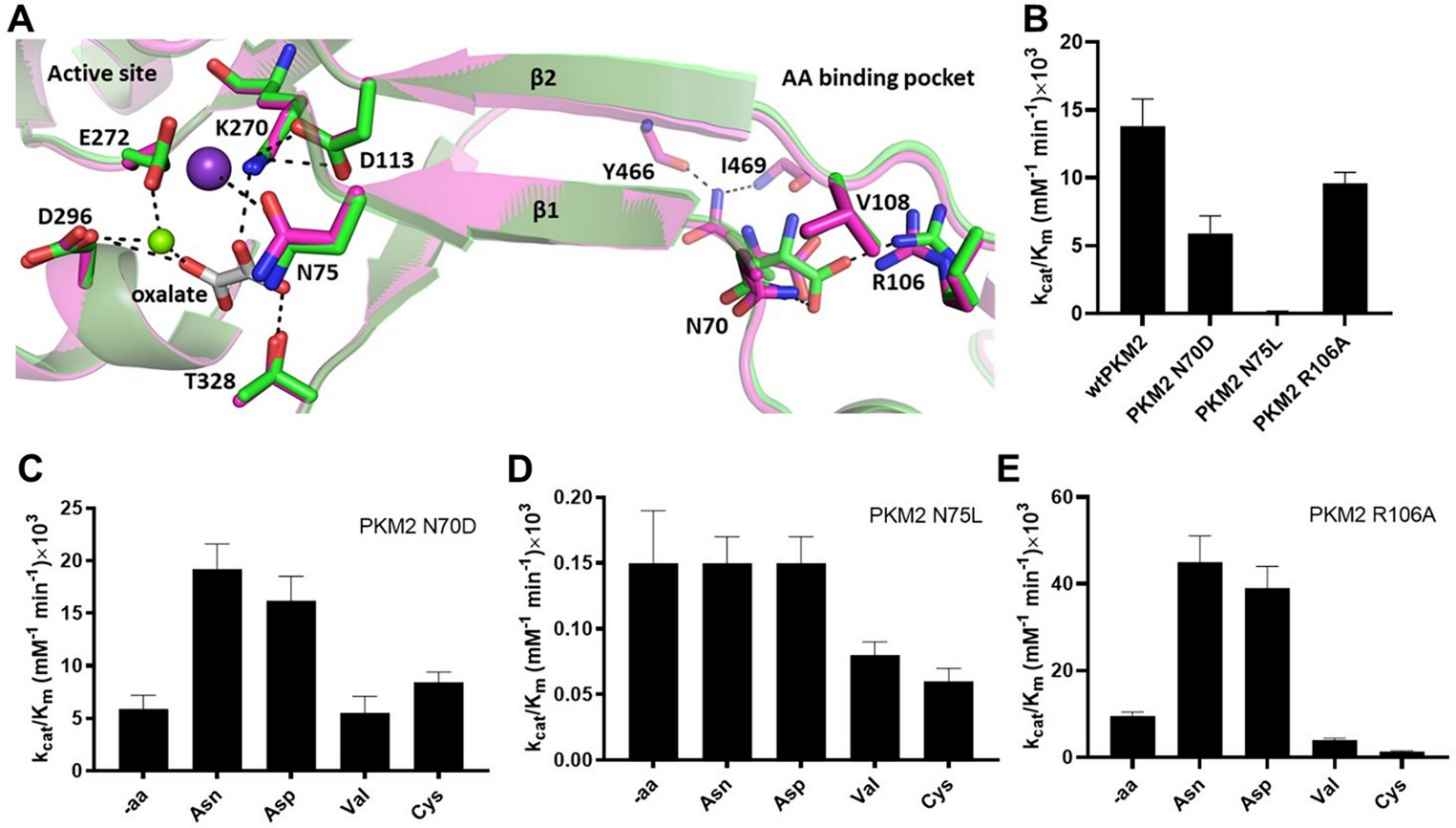
Proposed signal transfer pathway and differential effect of activating (Asn/Asp) and inhibiting AAs (Cys/Val) on PKM2 variants. (A) Proposal for long-range signal transduction pathway between the allosteric AA binding site (right) and active site (left) of PKM2, based on the crystal structures of PKM-Asn (magenta) and PKM2-Val (green). The two sites are connected by two β strands, β1 and β2. The violet and green spheres represent K^+^ and Mg^2+^, respectively. The dashed lines represent H-bonds. O, N and C are in red, blue, and backbone color, respectively. (B) Comparison of catalytic efficiencies (*k*_*cat*_*/K*_*m*_) of wtPKM2, PKM2 N70D, PKM2 N75L, and PKM2 R106A. wtPKM2 and PKM2 R106A show comparable activities, whereas PKM2 N70D and PKM2 N75L exhibit lower activity than wtPKM2. (C) The activity of PKM2 N70D increased in the presence of Asn/Asp, but Val/Cys do not impact the activity. (D) The *k*_*cat*_*/K*_*M*_ of PKM2 N75L is influenced in the presence of Val/Cys slightly but Asn/Asp have no effect. (E) The activity of PKM2 R106A is influenced by the activating and inhibiting AAs in a similar manner as that of wtPKM2, with *k*_*cat*_*/K*_*M*_ increasing with Asn/Asp, whereas Val/Cys inhibit the variant. The activity assays were performed with 65 nM PKM2 N70D, 4200 nM PKM2 N75L, and 50 nM PKM2 R106A. The concentration of PEP was varied between 0.025–8 mM and the ADP concentration was kept constant at 0.8 mM for all assays. The AA concentrations were fixed at 2 mM for all experiments, with the exception of PKM2 R106A, where 10 mM Val was used. The kinetic data were used to calculate the catalytic efficiencies. Kinetic parameters are listed in S1 Table.

On the basis of the crystal structure of cysteine bound complex of PKM2, we had proposed that two β-strands linking the active site and the AA binding site might be involved in long-range allosteric communication between the two sites [15]. To identify the residues involved in signal transduction, in the present study we altered N70 and N75 located at two ends of a β-strand (hereafter labeled as β1), connecting the AA binding site and the active site, respectively (Fig 1A). Specifically, N70, located near the AA binding site, was mutated to D (hereafter, PKM2 N70D) in order to break the interaction between the carboxylate oxygen of the AA ligand and sidechain -NH_2_ of N70 (Fig 1A).

Likewise, since R106 precedes the residues forming another β-strand (hereafter labeled as β2) and is involved in interacting with the carboxylate oxygen of all bound AA ligands [16, 17, 22], R106 was changed to A (hereafter, PKM2 R106A) to disrupt the H-bond interaction. In addition, N75, which is located near the active site and interacts with oxalate (an enolpyruvate analog [23]) via K^+^, water, and Mg^2+^ ions (PDB:1T5A) was altered to L (hereafter, PKM2 N75L) to disrupt the network of bonds between N75 and oxalate (S1C Fig). As the C = O group in the sidechain of N75 interacts with K^+^, a conservative mutation such as N75 to D75 would not break the electrostatic interaction (Fig 1A). Therefore, a non-conservative mutation, N75 to L75 was introduced in the present studies.

Biochemical and structural studies of wtPKM2 with Asn, Asp, Val [17], and Cys [15] have illustrated that the sidechain of activating AAs and the main chain of Val and Cys are responsible for transmitting the activating and inhibitory signal, respectively. In order to understand how the activation and inhibition signals are transmitted, we investigated PKM2 N70D, PKM2 N75L, and PKM2 R106A variants in the presence and absence of Asn, Asp, Val, and Cys. In general, our findings show that while Asn and Asp activated the PKM2 N70D variant by altering the oligomeric state of the variant from a mixture of tetramer and dimer/monomer equilibrium to predominantly a tetramer, the variant was not inhibited by Val/Cys. Interestingly the activity of PKM2 N75L remained unaltered in the presence of Asn/Asp, but Val/Cys inhibited it by shifting the oligomeric state from tetramer to a mixture of tetramer and dimer/monomer equilibrium.

Taken together, these results confirm that N70 and N75, along with β1 connecting these residues are part of the signal transduction pathway between the active site and the AA binding allosteric site. Furthermore, the results confirm that N70 is one of the residues responsible for transmitting the inhibitory signal. In addition, the data indicate that N75 is involved in the activation signal flow; however, it does not exclude the possibility of other residues involved in transmitting both activation and inhibition signals.

## Materials and methods

### Expression and purification of human PKM2

The human PKM2 gene was cloned in the pET28a vector and was gifted to us by M. G. Vander Heiden. The PKM2 mutant plasmids N70D, N75L, and R106A were generated by site-directed mutagenesis using mutagenic oligonucleotides. The following primers were used to generate each of the variants.

*PKM2 N70D*:

Forward primer (5’-3’): AGA TGA TTA AGT CTG GAA TGG ATG TGG CTC GTC TGA ACT

Reverse primer (5’-3’): AAG TTC AGA CGA GCC ACA TCC ATT CCA GAC TTA ATC ATC

*PKM2 N75L*:

Forward primer (5’-3’): TGG AAT GAA TGT GGC TCG TCT GCT ATT CTC TCA TGG AAC TCA TG

Reverse primer (5’-3’): TCA TGA GTT CCA TGA GAG AAT AGC AGA CGA GCC ACA TTC ATT CCA

*PKM2 R106A*

Forward primer (5’-3’): CCC CAT CCT CTA CGC GCC CGT TGC TGT G

Reverse primer (5’-3’): AGC AAC GGG CGC GTA GAG GAT GGG GTC A

For protein expression purposes, the plasmids were transformed into *E*. *coli* BL21(DE3) pLysS cells (Life Technologies, Grand Island, NY). Starter cultures prepared by inoculating single colonies of the mutant plasmids in 50 ml Luria-Bertani media were grown overnight at 37°C/180 rpm. 20–40 ml of the starter culture was used as inoculum in 1–2 L of Terrific Broth media and the culture was grown at 37°C/180 rpm till the optical density at 600 nm (OD_600_) reached 2.0. Subsequently 0.2 mM isopropyl β-D-1-thiogalactopyranoside was used to induce the cells and the culture was grown further for 20–22 hrs. at 22°C. The culture was centrifuged at 4,000 rpm for 30 mins at 4°C to harvest the cells. 20–40 gm of cell pellet was resuspended in 40–60 ml of 20 mM HEPES pH 7.5, 5 mM imidazole, 150 mM NaCl, and 5% glycerol (buffer A). The cell suspension was sonicated for 7–8 mins (8 secs ON, 20 secs OFF) for cell lysis and the cell debris was precipitated by centrifugation at 30,000 rpm for 30 min at 4°C. The 6×His-wtPKM2 and each PKM2 variants were purified by passing the supernatant through 10 ml of immobilized Ni-affinity resin pre-equilibrated with buffer A. The protein-bound column was washed with 70 ml of buffer A followed by 50 ml of buffer A containing 50 mM imidazole to remove the non-specifically bound protein. Finally, buffer A containing 450 mM imidazole was used to elute the pure protein and it was visualized by SDS-PAGE. The purified protein was dialyzed overnight in 20–50 mM Tris-HCl pH 7.5, 0.5 mM tris (3- hydroxypropyl) phosphine (THP), 150 mM KCl, 5% glycerol, and 0.5 mM EDTA (storage buffer) to remove the imidazole. The dialyzed protein was passed through HiLoad Superdex 200 16/600 (GE Healthcare Life science, Marlborough, MA) gel filtration column pre-equilibrated with storage buffer to remove any aggregated protein. To measure the protein concentration at 280 nm, a NanoDrop 2000c spectrophotometer (Thermo Scientific, Waltham, MA) with 1 mm pathlength was used. The MW and molar extinction coefficient (ε_280 nm_) were set at 59.96 kDa (corresponding to PKM2 monomer) and 29.91 M^-1^ mm^-1^, respectively. The readout was protein concentration expressed in mg/ml. The protein was stored in 100 μL aliquots at -80°C until use. For all experiments described hereafter, the protein concentration refers to monomeric PKM2.

### Pyruvate kinase activity assays

PKM2-lactate dehydrogenase (LDH) coupled assay was used to measure the activity of wtPKM2 and the PKM2 variants in the presence and absence of Asn, Asp, Cys, and Val using an Epoch microplate spectrophotometer (BioTek, Winooski, VT). Briefly, 10 nM, 65 nM, 4200 nM, 50 nM of wtPKM2, PKM2 N70D, PKM2 N75L, and PKM2 R106A were dissolved in a buffer containing 20 mM Tris-Cl pH 7.5, 150 mM KCl, 5 mM MgCl_2_, 4 units/ml LDH, and 0.5 mM NADH. 2 mM of Val/Asn/Asp/Cys were added to the enzyme solution and incubated on ice for 15 min (Note: The Val concentration used for PKM2 R106A was 10 mM). A varying concentration of PEP (0.025–8 mM) was used to initiate 100 μl reactions while keeping the ADP concentration fixed at 0.8 mM. The decrease in absorbance at 340 nm was recorded for a minute to monitor the progress of the reactions. Initial velocities were calculated from the rate of decrease of absorbance in GraphPad Prism (San Diego, CA) and were plotted against PEP concentration. The resulting curves were fitted using the Michaelis-Menten Eq (1),

$$Y=\frac{V_{max}\times X}{K_{m}+X}$$ (1)

where X is the substrate (PEP) concentration, *V*_*max*_ is the maximum velocity, Y is the initial velocity, and *K*_*m*_ is the Michaelis-Menten constant for PEP.

The kinetic data from PKM2 R106A in the presence of Cys was fitted using the allosteric sigmoidal Eq (2),

$$Y=\frac{V_{max}\times X^{h}}{K^{\prime }+X^{h}}$$ (2)

where X is the substrate (PEP) concentration, *V*_*max*_ is the maximum velocity, Y is the initial velocity, *h* is the Hill coefficient, and *K*’ is the Michaelis-Menten constant for PEP.

### Ligand binding assays

All ligand binding studies were conducted in a buffer containing 1.6 μM enzyme dissolved in 20 mM Tris-HCl (pH 7.5) and 150 mM KCl. Briefly, to measure the binding affinity of wtPKM2 and the variants for PEP/ADP in the absence and presence of Asp/Asn/Val/Cys, each enzyme solution was incubated for 15 min with 1 mM of corresponding AA dissolved in a buffer containing 5 mM MgCl_2_. The decrease of intrinsic tryptophan fluorescence with increasing substrate concentration was measured using a Cary Eclipse Fluorescence Spectrophotometer (Agilent Technologies, Santa Clara, CA). To determine the binding affinity of wtPKM2 and the variants for the AAs mentioned previously, the enzymes were dissolved in the buffer followed by monitoring fluorescence quenching as the AA concentration was increased. The wavelength used to excite PKM2 was set at 295 nm, while the emission wavelength was 340 nm with slit widths of 5 and 10 nm, respectively. The fluorescence intensity values were used to calculate fractional saturation, which was fitted to the one-site specific binding Eq (3), or the Hill Eq (4) using GraphPad Prism (San Diego, CA),

$$Y=\frac{B_{max}\times X}{K_{d}+X}$$ (3)

$$Y=\frac{B_{max}\times x^{h}}{K_{d}^{h}+x^{h}}$$ (4)

where *B*_*max*_ is maximum binding, Y is fractional saturation, *h* is the Hill coefficient, *K*_*d*_ is dissociation constant, and X is ligand concentration.

Since the *K*_*d*_ of PKM2 N70D for FBP without any AAs/for PEP in the presence of Asn/Asp, and the *K*_*d*_ of PKM2 R106A for PEP in the presence of Asp/Asn were lower than the enzyme concentration used, the fractional saturation was fitted using a ligand-binding model (5) as reported previously [21, 24]

$$f=f_{0}+{(f}_{m}-f_{0})\frac{(n\times P+x+K_{d})-\sqrt[{2}]{({n\times P+x+K_{d})}^{2}-4\times n\times Px}}{2\times n\times P}$$ (5)

where *f* is fluorescence signal resulting from AA binding to PKM2 R106A, *f*_*0*_ is the signal from PKM2 R106A in buffer solution, *P* and *x* total protein and added AA concentration, respectively, *f*_*m*_ is the maximum fluorescence intensity, *K*_*d*_ is dissociation constant, and *n* is the number of binding sites.

### Gel-filtration studies of wtPKM2 and PKM2 variants

A 0.1 mg/ml solution of wtPKM2 and each PKM2 variant in storage buffer with 10 mM THP (to regulate the redox state of the enzyme) was incubated for 15 min in the absence and presence of Asn/Asp/Val/Cys before injecting onto a Superdex 200 10/300 GL gel filtration column (24 ml, GE healthcare). The flow rate was kept constant at 0.5 ml/min using an AKTA Pure FPLC system (GE Healthcare Life Sciences, Marlborough, MA). All AA concentrations used in the experiments were fixed at 10 mM. Column calibration was conducted using gel-filtration molecular weight standards (Bio-Rad) containing bovine thyroglobulin (670 kDa), bovine γ-globulin (158 kDa), chicken ovalbumin (44 kDa), horse myoglobin (17 kDa), and vitamin B12 (1.35 kDa). The eluted protein peak was observed using UV absorbance at 280 nm. To normalize the lowest and highest intensity value, they were set to 0% and 100%, respectively, which was plotted against elution volume in GraphPad Prism (San Diego, CA).

### Crystallization of PKM2 N70D variant

PKM2 N70D was crystallized by mixing 10 mg/ml of the enzyme with 2 mM oxalate, and 5 mM MgCl_2_ in storage buffer followed by incubating the solution on ice at 4°C for 30 min. 1 μl each of the protein and precipitant solutions were mixed and set up for crystallization by sitting-drop vapor diffusion method at room temperature. Rod-shaped crystals of PKM2 N70D were obtained in a precipitant solution containing 0.2 M sodium malonate, 0.1 M bis-tris propane pH 7.0, and 22–24% PEG 3350. For cryopreservation, the crystals were looped and cleansed in a precipitant solution containing 25% (vol/vol) glycerol and mother liquor, followed by cryocooling in liquid nitrogen.

### Data collection and structure determination

X-ray diffraction data for PKM2 N70D crystals were collected using beamline 4.2.2 at Advanced Light Source (Berkeley, CA) using an RDI CMOS_8 M detector at 100K. The detector distance was fixed at 300 mm with an exposure time of 0.5 s and 0.2° oscillation. XDS package [25] and SCALA (CCP4) [26] were used for image processing, and the chain A of PKM2 S437Y (PDB:6B6U) was used as the search model for molecular replacement by PHASER [27]. PHENIX [28] and COOT [29] were used for iterative rounds of refinement and model building, respectively. Refinement strategies used in the initial rounds of refinement were restrained coordinate, rigid body, B-factor, occupancy, and simulated annealing. In the later rounds, simulated annealing and rigid body refinement were turned off, and water molecules were modelled into clear densities.

In all the four chains of PKM2 N70D, the first 13 residues, including the His-tag in the N-terminus was unmodelled due to the absence of electron density. The B domain was well modelled in all chains except in chain D, where residues 127–130 and 188–192 were missing. A portion (1–4 residues) of the FBP activation motif (FAM, residues 514–522) were missing in all the chains. In order to confirm that the FBP binding pocket of the variant lacked FBP, we fitted a complete FBP molecule in the pocket with partial occupancy and did an occupancy refinement. Interestingly, with an occupancy of ~0.7, a majority of the fitted region showed negative density except for a small portion of FBP, which included the phosphate group, thereby confirming the absence of FBP. The final model of the structure was verified by composite omit maps, and the structure was validated by the wwPDB validation server [30]. PyMol was used to generate the figures related to the structure [31]. The coordinates and structure factors of PKM2 N70D structure have been deposited in the protein data bank (PDB: 7L21). Data processing, refinement, and validation statistics are summarized in Table 1.

**Table 1:** X-ray data collection and refinement statistics.

	PKM2 N70D
PDB code	7L21
space group	C 2 2 2_1_
unit cell dimensions	* *
a b c (Å)	139.34 158.3 241.61
α β γ (deg)	90 90 90
Beamline	ALS 4.2.2
wavelength (Å)	1.000030
oscillation range (deg)	180
resolution range (Å)	63.81–2.29 (2.41–2.29)
no. of observations (no. of reflections)	1811940 (259830)
no. of unique reflections	119675 (17265)
Redundancy	15.1 (15.0)
CC1/2	0.999 (0.940)
*R*pim	0.034 (0.166)
completeness (%)	100.0 (100.0)
*I*/σ(*I*)	22.9 (4.9)
*R*merge (%)	12.4 (60.3)
*R*meas (%)	13.3 (64.7)
Wilson B factor (Å^2^)	24.39
**Refinement**	
R_work_ (%)	18.2 (20.2)
R_free _(%)	22.8 (25.8)
no. of reflections	119610
no. of molecules per asu	4
no. of atoms	
Proteins	15301
Ligands	119
Water	664
average B-factor (Å)	
macromolecules	29.79
Ligands	33.84
Water	28.57

## Results

### Activities of PKM2 variants decreased relative to wtPKM2

To understand how the PKM2 variants described in this study impact its pyruvate kinase activity, assays were performed with wtPKM2 and PKM2 R106A, N70D, and N75L using varying concentrations of PEP keeping ADP concentration fixed. The results indicate that the catalytic efficiencies (*k*_*cat*_*/K*_*m*_) of wtPKM2 (13.8±2 ×10^3^ mM^-1^ min^-1)^ and PKM2 R106A (9.6±0.8 ×10^3^ mM^-1^ min^-1^) are quite similar (Fig 1B, S1 Table). While the turnover number (*k*_*cat*_) of PKM2 R106A (4.9 min^-1^) decreased slightly compared to wtPKM2 (*k*_*cat*_ = 9.7±0.4 min^-1^), it is very similar to that of N70D (*k*_*cat*_ of 4.6 min^-1^) (S1 Table). The *k*_*cat*_*/K*_*m*_ of PKM2 N70D (5.9±1.3 ×10^3^ mM^-1^ min^-1^) decreased compared to wtPKM2 or PKM2 R106A (Fig 1B, S1 Table), indicating that alteration of residues in the AA binding pocket decreased the activity only slightly.

In contrast, mutation of N75 to the hydrophobic leucine residue significantly decreased the turnover number (0.012±0.001 min^-1^) relative to wtPKM2 (9.7±0.4 min^-1^). Interestingly, the *K*_*m*_ of PEP for N75L (0.08 mM) is better than wtPKM2 (0.7 mM). These results indicate that mutation of N75, which is located at the active site, has a greater impact on the *k*_*cat*_ and therefore an overall reduction in the catalytic efficiency of PKM2 N75L was observed (Fig 1B, S1 Table). These results indicate that PKM2 N70D and PKM2 N75L variants have decreased activities compared to wtPKM2, whereas the activity of PKM2 R106A is comparable to wtPKM2.

### N70 and N75 are involved in signal transmission between AA binding site and the active site of PKM2

The catalytic efficiency of wtPKM2 has been demonstrated to be influenced by activating (Asn/Asp) and inhibitory AAs (Val/Cys) [17]. To understand how the mutation of residues connecting the AA binding site with the active site of PKM2 influence the allosteric regulatory properties of activating and inhibiting AAs, pyruvate kinase assays were performed with the PKM2 variants (including wtPKM2 as positive control) in the presence and absence of Asn, Asp, Val, and Cys (Fig 1C–1E, S2 Fig, S1 Table). The results show that in contrast to wtPKM2, PKM2 N70D was not inhibited by Val/Cys (*k*_*cat*_*/K*_*m*_ of 5.9±1.3×10^3^ mM^-1^ min^-1^, -Val/Cys vs. 5.5±1.6/8.4±1.0×10^3^ mM^-1^ min^-1^, +Val/Cy) (Fig 1C, S2A–S2C Fig, S1 Table). However, Asn/Asp were able to slightly activate PKM2 N70D (*k*_*cat*_*/K*_*m*_ = 5.9±1.3×10^3^ mM^-1^ min^-1^ -activators vs. ~17×10^3^ mM^-1^ min^-1^ +activators) (Fig 1C, S2C Fig, S1 Table), indicating that a change to the acidic D decreased the magnitude of activation relative to wtPKM2. These results illustrate that N70 is involved in transmitting the inhibitory signal and the activation signal is getting transmitted through some other unknown residues.

In contrast, an alteration of N75 (located near the active site and on another end of β1) to the hydrophobic leucine resulted in an opposite response of PKM2 N75L to the AAs compared to the PKM2 N70D. In particular, the presence of Val/Cys decreased the *k*_*cat*_*/K*_*m*_ of PKM2 N75L by two-fold (0.15±0.04×10^3^ mM^-1^ min^-1^ -Val/Cys vs. 0.08±0.01/0.06±0.01×10^3^ mM^-1^ min^-1^ +Val/Cys) (Fig 1D, S1 Table). However, in the presence of Asn/Asp, the *k*_*cat*_*/K*_*m*_ of PKM2 N75L was ~0.15×10^3^ mM^-1^ min^-1^, which is similar to that without the activators, thus indicating an absence of activation (Fig 1D, S2D Fig, S1 Table). These results indicate that N75 is involved in activating signal transmission and its alteration does not influence inhibitory signal transmission.

Interestingly, PKM2 R106A responds to AA-mediated activation and inhibition in a manner similar to wtPKM2 (Fig 1E, S2A, S2B and S2E Fig) [17]. In the presence of Asn and Asp, the catalytic efficiencies of R106A increased from 9.6±0.8×10^3^ mM^-1^ min^-1^ (- activating AAs) to 45±6×10^3^ mM^-1^ min^-1^ (+Asn) and 39±5×10^3^ mM^-1^ min^-1^ (+Asp) (Fig 1E, S2E Fig, S1 Table). Likewise, in the presence of Val and Cys, the catalytic efficiencies of PKM2 R106A decreased by ~2–7 fold (Fig 1E, S2E Fig, S1 Table).

Taken together, the above results indicate that R106 is not involved in transmitting the allosteric signal from the AA binding site to the active site of PKM2, whereas N70 and N75, located on either end of β1, are key residues involved in signal transmission. In particular, the differential and reverse effect of AAs on the activities of PKM2 N70D and N75L confirms that N70 and N75 are involved in transmitting the inhibition and activation signal, respectively.

### Impact of PKM2 variants on the substrate binding affinities

**Fig 2:**
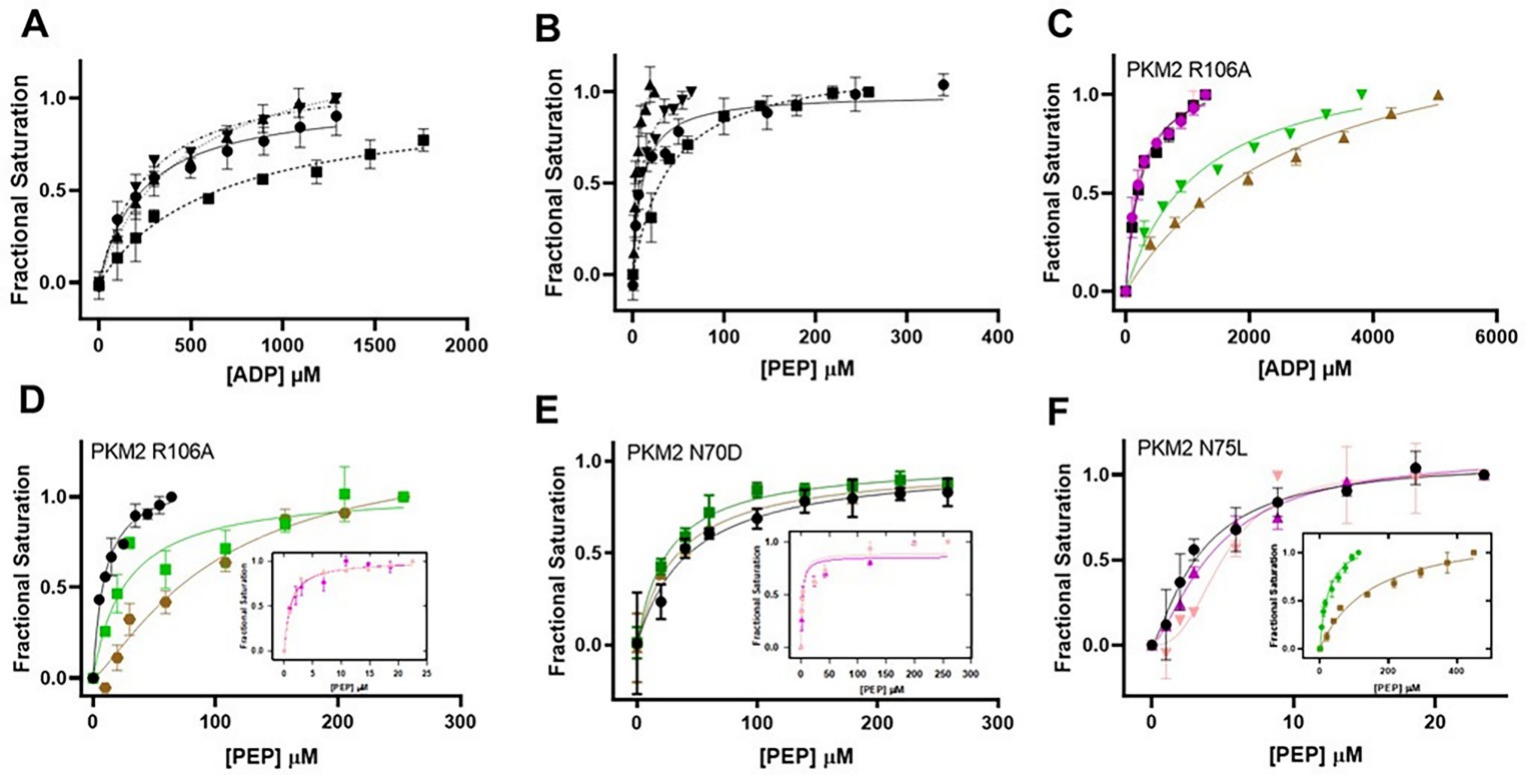
Determination of substrate binding affinity of PKM2 variants. (A) Binding of ADP and (B) PEP to wtPKM2 (-●-), PKM2 N70D (—■—), PKM2 N75L (⸱⸱▲⸱⸱), and PKM2 R106A (⸱⸱−▼⸱⸱−). The ADP and PEP binding affinity of PKM2 N70D is weaker than that of wtPKM2 and other variants. The PEP binding affinity of PKM2 R106A is similar to wtPKM2, and that of PKM2 N75L is slightly tighter than wtPKM2. (C) ADP binding studies of PKM2 R106A in the absence (black) and presence of Asn (magenta), Asp (salmon), Val (green), and Cys (brown). The ADP binding affinity of the R106A variant decreases in the presence of Val and Cys, while Asn and Asp do not affect it. (D-F) PEP binding affinities of (D) PKM2 R106A (E) PKM2 N70D (F) PKM2 N75L in the absence and presence of the AAs (color scheme for the AAs is identical to (C)). The PEP binding affinity of PKM2 R106A increased in the presence of Asn/Asp (inset in D) and decreased upon incubation with Val/Cys. The PEP binding affinity of PKM2 N70D increased in the presence of Asn and Asp (inset in E), while Val/Cys did not affect the *K*_*d*_. For PKM2 N75L, Val and Cys decrease its PEP binding affinity (inset in F), while Asn/Asp have no effect. The concentration of each enzyme used in all experiments were 1.6 μM and all the AA concentrations were constant at 1 mM. The *K*_*d*_ values are listed in S2 Table.

To understand how the mutation of the specific residues described here impact the substrate binding affinities, fluorescence studies were performed with wtPKM2, PKM2 N70D, R106A, and N75L using varying concentrations of PEP or ADP and monitoring changes in the intrinsic tryptophan fluorescence (Fig 2A and 2B, S2 Table). The results indicate that the ADP binding affinity of PKM2 R106A (*K*_*d*_ *=* 257±24 μM) is similar to wtPKM2 (*K*_*d*_ *=* 237±54 μM) [17]. Likewise, the PEP binding affinities of PKM2 R106A (9.5±1.1 μM) and wtPKM2 (10±2 μM) are similar. The similarity in the substrate binding affinities of PKM2 R106A and wtPKM2 are in agreement with their similar activities (Figs 1B, 2A and 2B, S2 Table).

With PKM2 N70D, both ADP (*K*_*d*_ = 644±154 μM) and PEP (*K*_*d*_ = 40.3±5.1 μM) binding affinities are weaker compared to wtPKM2, which is consistent with its decreased activity (Figs 1B, 2A and 2B, S2 Table). Interestingly for PKM2 N75L, ADP binding affinity (*K*_*d*_ = 449±48 μM) is relatively weak compared to wtPKM2, but it has a 3-fold tighter affinity for PEP (*K*_*d*_ = 3.5±0.8 μM). The greater affinity of PKM2 N75L for PEP is in agreement with its low *K*_*m*_ value for PEP. These results demonstrate that PKM2 N70D and N75L influence the binding affinities for PEP and ADP, whereas PKM2 R106A exhibits substrate binding affinities similar to that of wtPKM2.

### Differential effect of activating and inhibiting AAs on the substrate binding affinities of PKM2 variants

To decipher how activating (Asn/Asp) and inhibitory (Cys/Val) AAs influence the ADP and PEP binding affinities of the PKM2 variants, ligand binding studies were carried out using fluorescence changes. In previous studies with wtPKM2, we have demonstrated that the ADP binding remains unaltered (*K*_*d*_ ~0.2–0.6 mM) in the presence of Asn/Asp/Val [17] and Cys [15] (S3A Fig, S2 Table). Likewise, in the present study, the affinities of PKM2 N70D and N75L for ADP remained unchanged in the absence or presence of the AAs (*K*_*d*_ ~0.3–0.7 mM) (S3C and S3D Fig, S2 Table).

With PKM2 R106A, the ADP binding affinity in the presence of Val (*K*_*d*_ ~1.2 mM)/Cys (*K*_*d*_ ~ 2.9 mM) was ~10-fold weaker than that in the absence of any AAs (*K*_*d*_ = 257±24 μM) (Fig 2C, S2 Table). However, in the presence and absence of Asn/Asp, the ADP binding affinities of PKM2 R106A remain unchanged (*K*_*d*_ ~200–250 μM) and are similar to that of wtPKM2 (*K*_*d*_ ~200–500) (Fig 2C, S2 Table) [17]. The PEP binding affinity of PKM2 R106A in the presence of activator AAs (Asn/Asp) improved, with *K*_*d*_ changing from ~9.5 μM (-AA) to ~1 μM (+Asn/Asp). In contrast, in the presence of Val/Cys, the PEP binding affinity of PKM2 R106A weakened (*K*_*d*_ ~26–100 μM) (Fig 2D, S2 Table). A similar pattern of altered affinity for PEP was observed previously with wtPKM2 using Asn, Asp, Val [17], and Cys (S3B Fig), with increased and decreased affinity in the presence of activating (Asn, Asp) and inhibitory AAs (Val or, Cys), respectively (S2 Table). The influence of these AAs on the PEP binding affinity of PKM2 R106A is also consistent with the activity assays showing that Asn/Asp regulates the activity by improving the PEP binding affinity (Figs 1E and 2D, S2 Table).

Interestingly, the PEP binding affinity of PKM2 N70D remained unaltered in the presence of either Val/Cys (*K*_*d*_ ~40 μM without AA vs. ~22–41 μM with Val/Cys) (Fig 2E, S2 Table). However, the presence of either Asn/Asp enhanced the binding affinity of the variant for PEP consistent with the increased activity with Asn/Asp, and as observed previously with wtPKM2 (Figs 1C and 2E, S2 Table) [17].

The PEP binding affinity of PKM2 N75L remained unaltered in the presence of Asn/Asp (*K*_*d*_ ~3–5 μM in the absence and presence of the AAs), which is consistent with the activity assay results with Asn/Asp (Figs 1D and 2F, S2 Table). However, the presence of Val/Cys decreased the PEP binding affinity of PKM2 N75L (*K*_*d*_ ~3.5 μM without AA vs. ~24–152 μM with Val/Cys), again consistent with their slight inhibition effect (Figs 1D and 2F, S2 Table). The differential effect of the AAs on the PEP binding affinity of PKM2 N75L is in agreement with the response in activity of the variant in the presence of various AAs (Figs 1D and 2F).

### The AA binding affinity of PKM2 variants

**Fig 3:**
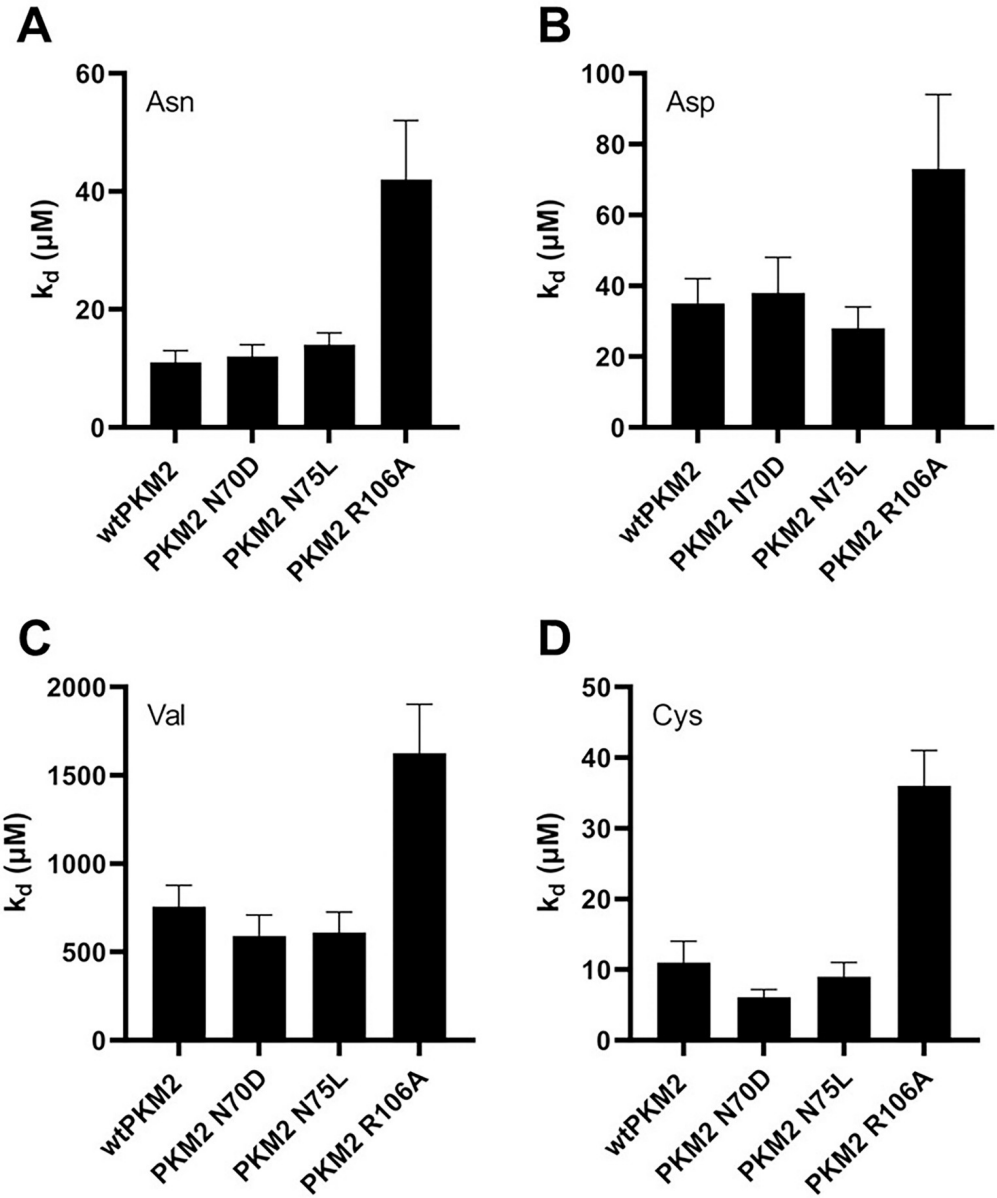
Binding affinities of PKM2 N70D, PKM2 N75L, PKM2 R106A, and wtPKM2 for: (A) Asn, (B) Asp, (C) Val, and (D) Cys. The data demonstrate that the R106A variant has a much weaker binding affinity for all AAs. The concentrations of each enzyme were kept constant at 1.6 μM. The dissociation constant (*K*_*d*_) values are listed in S3 Table.

To gain insight into how alteration of the residues in the AA binding pocket (N70D and R106A) and the active site (N75L) influence the AA binding affinities of the variants, ligand binding studies were conducted using different AAs (Asn, Asp, Val, and Cys) as titrants (Fig 3, S3 Table). The binding affinities of PKM2 N70D for the AAs were similar to that of wtPKM2, indicating that changing the charge in N70 did not alter the binding affinity for all the AAs tested. Likewise, the affinities of PKM2 N75L for various AAs were very similar to wtPKM2. In contrast, the affinity of PKM2 R106A for Asn (*K*_*d*_ = 42±10 μM), Asp (*K*_*d*_ ~70 μM), Val (*K*_*d*_ ~1600 μM), and Cys (*K*_*d*_ = 36 μM) decreased by ~2–4 fold relative to wtPKM2, indicating that the mutation significantly impedes AA binding (Fig 3, S3 Table). As expected from the crystal structure, this result confirms that R106 is responsible for anchoring the AAs in the amino acid binding site.

### Oligomeric states of the PKM2 variants alter in the presence of activating and inhibitory AAs

**Fig 4:**
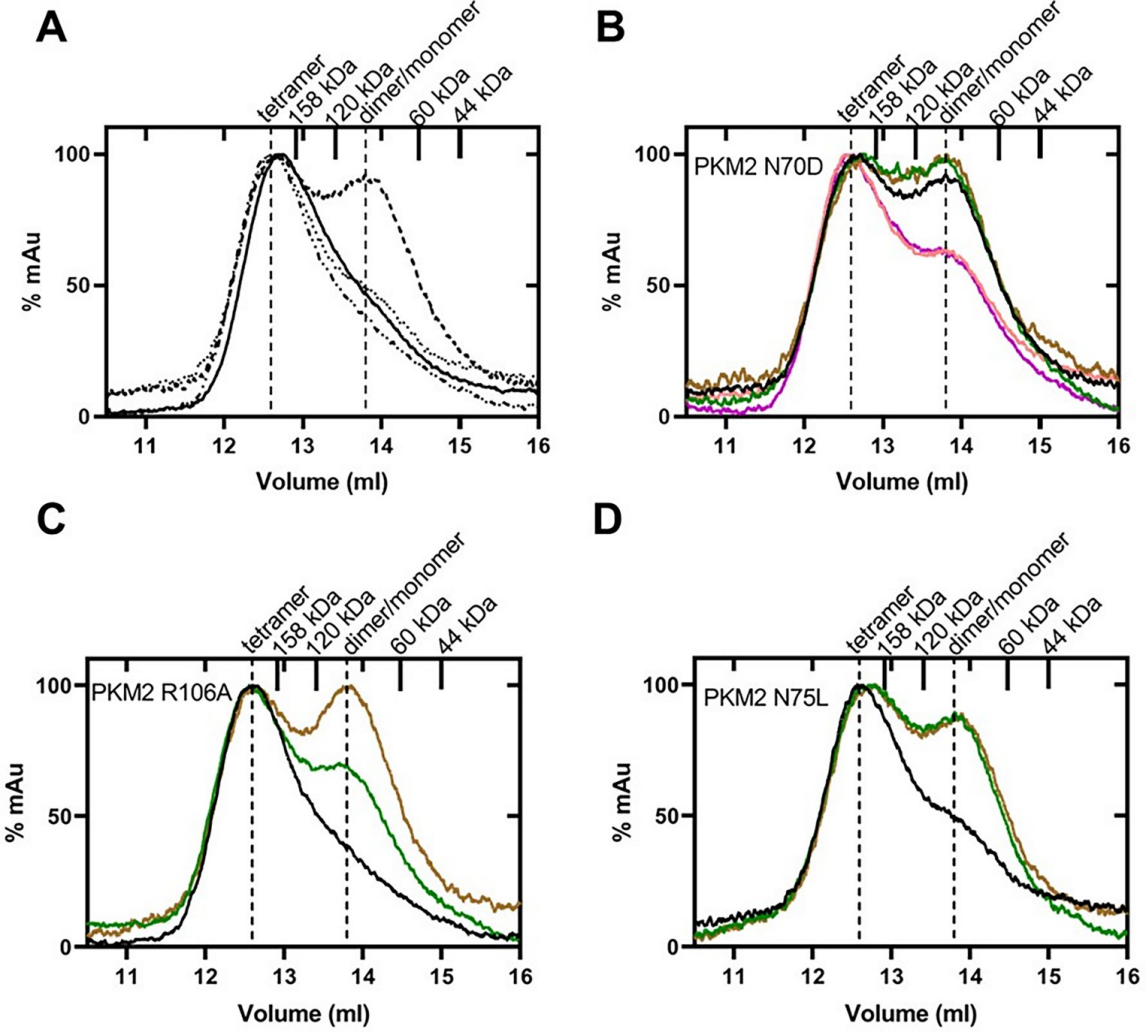
Gel filtration chromatograms of wtPKM2 and PKM2 variants in the absence and presence of AAs demonstrating the differential effect of AAs. (A) The profiles show that PKM2 N70D (—) exist as a clear mixture of tetramer and dimer/monomer equilibrium, wtPKM2 (−)/PKM2 R106A (⸱⸱−) exist as tetramers, and PKM2 N75L (⸱⸱⸱) has some dimer/monomer fraction. (B) The oligomeric state of PKM2 N70D becomes predominantly tetrameric upon incubation with Asn/Asp but remains as an equilibrium of tetramer and dimer/monomer mixture with Val/Cys (green/brown trace). (C) PKM2 R106A and (D) PKM2 N75L exist primarily in the tetrameric state and upon addition of Val/Cys shift to an equilibrium mixture of tetramer and dimer/monomer. The standards (158 kDa and 44 kDa) and theoretical molecular weight of dimer (120 kDa) and monomer (60 kDa) are marked as ticks on the upper axis. Colors: Chromatograms of PKM2 variants in the absence (black) and presence of Asn (magenta), Asp (salmon), Val (green), and Cys (brown). The concentrations of each enzyme and AAs were kept constant at 0.1 mg/ml and 10 mM, respectively.

To understand if the oligomeric state of PKM2 variants is impacted upon mutation, size-exclusion chromatography (SEC) was carried out and the results indicate that both PKM2 R106A and N75L exist mostly as a tetramer similar to that of wtPKM2 (Fig 4A). In contrast, the chromatogram of PKM2 N70D shows a clear mixture of tetramer and dimer/monomer equilibrium in the absence of any AAs (Fig 4A), indicating somehow mutation alone is perturbing its oligomeric state, which is also consistent with its decreased activity (Fig 1B) and substrate binding affinities (Fig 2A and 2B).

With wtPKM2, it was demonstrated that the activating (Asn/Asp) and inhibitory AAs (Val/Cys) regulate the activity of PKM2 by altering the oligomeric state [17]. To gain insight into how these AAs influence the oligomeric state of the PKM2 variants, gel filtration studies were conducted in the presence and absence of various AAs. With PKM2 N70D, the dimer/monomer fraction decreased in the presence of Asn/Asp, and an increase in the tetrameric active PKM2 N70D population was observed (Fig 4B, black vs. magenta/salmon trace). In contrast, the addition of Val/Cys to PKM2 N70D did not change the oligomeric state of the variant (Fig 4B, green and brown traces). The influence of AAs on the oligomeric state of PKM2 N70D directly correlates with its activity in the presence of these AAs (Fig 1C).

The gel filtration profile of PKM2 R106A in the presence of the inhibitory AAs (Val/Cys) resulted in a mixture of tetramer and dimer/monomer equilibrium (Fig 4C), similar to that observed with wtPKM2 [15, 17]. In the absence of any AAs, PKM2 N75L exists mostly as a tetramer with some dimer/monomer equilibrium fraction (Fig 4D, black line). Upon incubation with Val/Cys, the dimer/monomer fraction increased, which confirms that both of these AAs inhibit PKM2 N75L by shifting the oligomeric state (Figs 1D and 4D). Such a shift from an active tetramer to a mixture of tetramer and dimer/monomer equilibrium has been reported with wtPKM2 in the presence of Val [17] and shown here with Cys (S4 Fig). Considering both PKM2 N75L and PKM2 R106A mostly exist in the tetrameric state, the effect of activating AAs on the oligomeric state was not investigated as the gel filtration profiles are expected to resemble the tetramer. This assumption is on the basis of previous studies with predominantly tetrameric wtPKM2 and activators, where no change in oligomeric state was observed [17].

### Crystal structure of PKM2 N70D unravels a 3-way allosteric signal communication between the active site and the AA- and FBP- binding sites

#### Overall structure of PKM2 N70D

To gain insight into how mutation of specific residues in either end of β1 (N70D and N75) regulate the activity and oligomeric state differently from that of wtPKM2, the crystal structure of PKM2 N70D was determined. Attempts to determine the structures of PKM2 N75L and PKM2 R106A were unsuccessful. PKM2 N70D (PDB: 7L21) was crystallized in the C 2 2 2_1_ space group at 2.29 Å, and it consists of four molecules in the asymmetric unit (asu). An alignment of the structure of PKM2 N70D with wtPKM2-FBP (PDB:1T5A) showed that the overall tetramers are similar, with negligible difference in the orientation of their subunits (S1A Fig).

Each PKM2 N70D monomer consists of all four known domains. Structural alignment of a monomer, including the B-domain (chain A) of PKM2 N70D with wtPKM2-FBP (PDB:1T5A, with 458 C_α_ atoms) and PKM2-Cys (PDB:6NUI, with 350 C_α_ atoms), resulted in root mean square deviations (rmsd) of 0.30 Å and 0.381 Å respectively, indicating that the structures are similar (S1B Fig). The B-domain of PKM2 has been reported to be flexible in general [5, 32]. However, the B-domain in all four chains of PKM2 N70D aligned well with wtPKM2-FBP (S1 Fig). The overall architecture of the active site within the A-domain is similar to wtPKM2-FBP and PKM2-Cys (PDB:6NU1) and consists of one molecule of oxalate and Mg^2+^ ion in all four chains. (S1B Fig). While several studies have reported FBP to co-purify and co-crystallize with wtPKM2 even without adding it in the crystallization condition [7, 15, 17], in PKM2 N70D structure only a portion of unbound FBP molecule (comprising of the phosphate moiety) was present at an occupancy of ~0.7 in all four chains. The lack of entire FBP in the C-domain and positioning of the phosphate group of FBP into the electron density was verified as described in the methods section.

#### Movement of key residues in the AA binding pocket, FBP-binding site, and interface of PKM2 N70D

**Fig 5:**
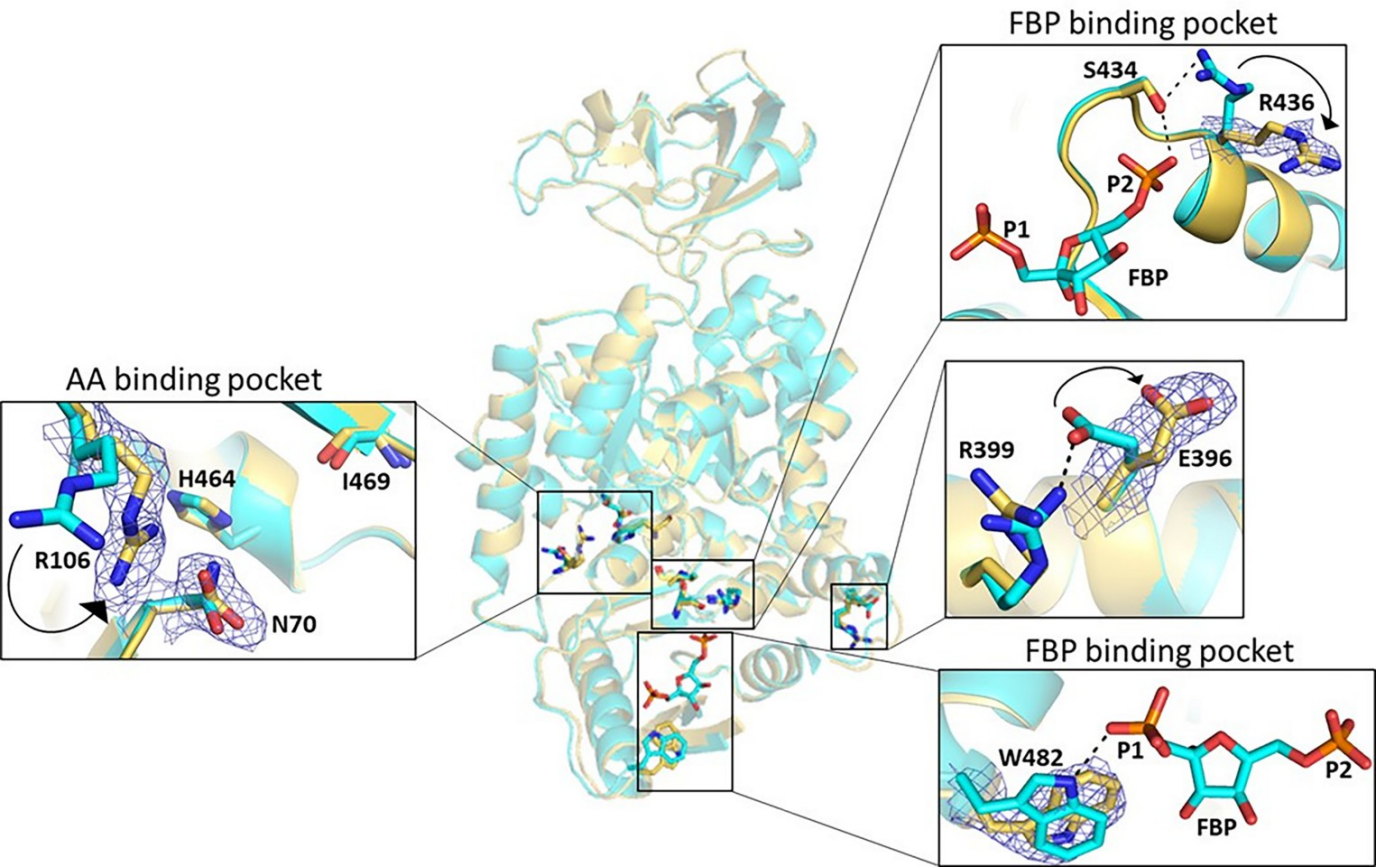
Crystal structure of PKM2 N70D. Superimposition of the AA binding pocket and FBP binding pocket of PKM2 N70D (yellow, PDB: 7L21) and wtPKM2 (cyan, PDB:1T5A) displays structural changes. Left inset: R106 in the AA binding pocket of PKM2 N70D (chain B) showed a shift compared to wtPKM2. D70 superimposed well with N70 of wtPKM2. Top right inset: R436 in the FBP binding pocket of PKM2 N70D (chain D) flipped outwards compared to wtPKM2, thereby breaking the bond with S434, which in turn is connected to FBP. Center right inset: E396 on the monomer interface of PKM2 N70D (chain A) moved outwards, breaking the H-bond with R399. Bottom right inset: W482 in the FBP binding pocket (chain A) rotates by 180° along the C_β_-C_Ɣ_ bond compared to wtPKM2, which results in breakage of H-bond between the indole ring and FBP. Arrows show the movement of AA sidechains. H-bonds are indicated by dashed lines. O, N, P, and C atoms are in red, blue, orange, and backbone color, respectively. Composite omit 2*F*_*o*_*—F*_*c*_ maps (blue mesh) contoured at 1.0 *σ* were generated for residues D70, R106, R436, E396, and W482.

The AA binding pocket of PKM2 N70D did not undergo significant structural changes by replacing the polar sidechain with an acidic group. A close-up view of the AA binding site displayed a shift of R106 compared to wtPKM2, which might be due to the flexible nature of the arginine side chain (Fig 5, left inset).

Interestingly, significant movements of various residues occurred in the FBP-binding pocket of PKM2 N70D (Fig 5, right insets). In particular, residues that interact with the phosphate groups of FBP in wtPKM2-FBP structure (PDB:1T5A), such as R436, W482 were shifted in PKM2 N70D. In both chains C and D, the sidechain of R436 flips out and away from the FBP-binding site, thus breaking its contact with S434, which is involved in H-bond interaction with P2 phosphate of FBP (Fig 5, top inset). Due to weak electron density, R436 was unmodeled in chains A and B. The sidechain of W482, in chain A, rotates by 180° about the C_β_-C_Ɣ_ bond thus disrupting the H-bond with P1 phosphate of FBP (Fig 5, bottom inset). However, no such rotation was observed in other chains. The sidechain of K433, that is known to be involved in interacting with the P1 phosphate of FBP [20], could not be modeled in all chains due to lack of electron density. Such structural changes involving the FBP-binding residues and the disruption of critical interactions with FBP were observed in FBP-free structures of PKM2 variants, such as PKM2 S37E and PKM2 S37D reported by us recently [20]. Finally, the sidechain of E396 located at the dimer-dimer interface swings away, breaking the H-bond with R399 (Fig 5, middle right inset). Similar movements of these interface residues were observed in our recent structures with phosphoserine- and acetyl-lysine- mimetic variants of PKM2 [20].

To understand if the N70D mutation in the AA binding pocket altered the FBP binding affinity of the variant, fluorescence quenching studies were carried out. The affinity of PKM2 N70D for FBP (*K*_*d*_ ~1.9768±0.0002 nM) was slightlylower than wtPKM2 (*K*_*d*_ = 0.98±0.1 nM) [20] (S5 Fig), which indicates that the ~20Å distance between the AA binding site and the FBP binding pocket is enough for the N to D mutation to cause a long-range effect leading to structural changes in the FBP site, thus impacting interactions with FBP.

## Discussion

Regulation of PKM2 by different small molecules and metabolites plays a vital role in maintaining the growth and proliferation of cancer cells. Various AAs bind to the AA binding pocket of PKM2 and likely work in tandem to assist in cancer cell proliferation by modulating the activity of PKM2 [15–17, 22]. It has been shown that AAs with polar functional groups such as Ser/Asn/Asp are similarly anchored in the AA binding pocket and activate PKM2 by converting into a pure tetrameric state [16, 17]. In contrast, Cys/Val with nonpolar sidechains inhibit PKM2 by shifting the active tetramer to a less active, dimer/monomer state [15, 17]. Likewise, other inhibitory AAs such as phenylalanine or tryptophan inhibit PKM2 by changing the conformation from an active, tetrameric, relaxed state to an inactive, tensed state [22]. However, the residues involved in transferring the allosteric signals have not yet been mapped. In the present study, we sought to identify key residues involved in the allosteric signal communication from the AA binding pocket to the active site.

On the basis of crystal structures of PKM2-Asn/Asp [17], PKM2-Cys [15], and PKM2-Ser [16], various residues (I469, G46, N44, and N70) were identified to form a network and interact with the bound AA ligand (Fig 6 of [17]. A closer inspection of the activating and inhibitory AA-bound complexes of wtPKM2 exposed that N70 and I469 were involved in direct H-bond interactions with the bound AAs (Figs 5, 6 of [17]. In particular, the sidechain -NH_2_ group of N70 interacts with the mainchain carboxylate atom of all bound AAs, and its sidechain carbonyl (-C = O) is also involved in other interactions within the AA binding pocket [15, 17, 22]. Likewise, the backbone carbonyl of I469 interacts with the main chain -NH_2_ group of most AAs, with the exception of bound Asn/Asp that interacts with I469 via their sidechains [17]. As N70 resides at one end of the β1 that connects the AA binding site to the active site, we hypothesized that it might be involved in allosteric signal transmission from the AA binding site. To perturb the interaction with AAs, the polar neutral sidechain of N70 was converted to the polar acidic group, generating the PKM2 N70D variant.

Interestingly, activity assays with PKM2 N70D and the inhibitory AAs reveal that Val/Cys does not inhibit the variant (*k*_*cat*_*/K*_*m*_ ~6×10^3^ mM^-1^ min^-1^, -AA vs. *k*_*cat*_*/K*_*m*_ ~5×10^3^/8×10^3^ mM^-1^ min^-1^, +Val/Cys, respectively) (Fig 1B and 1C, S1 Table). Likewise, binding studies with the variant showed that Val/Cys does not alter the affinity of PKM2 N70D for PEP (*K*_*d*_ ~ 21–40 μM, +/-Val/Cys). In sync with the unchanged activity and PEP binding affinity of the variant in the presence of Val/Cys, the oligomeric state of PKM2 N70D also remains unchanged in the presence of the AA inhibitors (Fig 4B). These results are contrary to that of wtPKM2, where Val/Cys inhibited the enzyme by decreasing the PEP binding affinity (Fig 2E, S2 Table) [15, 17]. In contrast, the impact of activating AAs on PKM2 N70D is very similar to that of wtPKM2. In the presence of Asn/Asp, the affinity of PKM2 N70D for PEP is tighter (*K*_*d*_ ~2 μM, +Asn/Asp vs. ~40 μM, -AA) (Fig 2E, S2 Table), with a concomitant increase in activity (Fig 1C, S1 Table). The increased activity of PKM2 N70D and its decreased binding affinity for PEP in the presence of AA activators agree well with the change in oligomeric state from an equal mixture of tetramer and dimer/monomer (-AA) to predominantly tetramer with some dimer/monomer fraction (+Asn/Asp) (Fig 4B). Finally, the N70D mutation alone does not influence the binding of AAs to the variant as the affinity of PKM2 N70D for all AAs studied remained nearly similar to that of wtPKM2 (S3 Table). Taken together, the data effectively demonstrates that mutation of N70 breaks the flow of the inhibitory signal from the AA binding pocket to the active site without altering the binding of the AAs, thus confirming that N70 is involved in transmitting Val/Cys mediated inhibitory signals. The results with PKM2 N70D further provide a clue that there is an alternate route or residues involved in transmitting the activation signal from the AA binding site.

An interesting aspect of PKM2 N70D that is worth mentioning is that the mutation alone decreased the activity compared to wtPKM2, and the variant appeared as a mixture of tetramer and dimer/monomer equilibrium in solution (Figs 1B, 4A and 4B). Whereas wtPKM2 mostly exists in the tetrameric state as shown here (Fig 4A) and in previous studies [15, 17], with FBP being responsible for maintaining it in the tetrameric state [5]. The FBP binding affinity of PKM2 N70D (*K*_*d*_ = 1.9768±0.0002 nM) is rather close to wtPKM2 (*K*_*d*_ = 1 nM [20], which in theory makes sense as changing a residue in the AA binding site would not alter the affinity for FBP in the C-domain. However, the crystal structure of PKM2 N70D clearly displayed structural changes in the FBP binding pocket located ~22 Å away from the AA binding site (Fig 5, S1B Fig). In particular, significant movement of residues W482 (involved in FBP binding), R436 (involved in FBP mediated allosteric signal communication), and E396 (involved in FBP dependent PKM2 activation) occurred, disrupting key interactions that are otherwise present in wtPKM2-FBP (PDB:1T5A) (Fig 5). Similar structural changes in the FBP-binding site were observed previously for PKM2 K433Q, PKM2 S37D, and PKM2 S37E [20]. Furthermore, the side chain of K433, an important FBP binding residue, was unmodeled in PKM2 N70D due to poor electron density. Thus the movement of key residues results in disruption of various electrostatic and H-bond interactions with FBP, leading to the eviction of FBP from its binding pocket, consequently perturbing the PKM2 N70D tetramer to a mixture of tetramer:dimer/monomer states (Figs 4A, 4B and 5). The crystal structure of PKM2 N70D evidently unravels a 3-way allosteric signal communication involving the AA- and FBP- binding sites and the active site.

We had previously demonstrated using ligand binding studies in the presence of Cys/Ser [15], or Asn/Asp/Val [17] and PEP and/or, Mg^2+^ that a functionally relevant bidirectional coupling mechanism existed between the AA binding site and the active site of PKM2 [17]. Considering that oxalate (an analogue of enolpyruvate) along with active site residues (E272, D296, and N75) are associated with binding Mg^2+^ and K^+^ ions and given N75 is located at one end of β1, we hypothesized N75 is likely involved in transmitting an allosteric signal between the AA binding site and the active site of PKM2 via β1. To disrupt this potential signal communication route, the polar N75 residue was changed to the nonpolar L generating the PKM2 N75L variant. Interestingly, PKM2 N75L exhibited opposite outcomes from that of PKM2 N70D as a change in N to D in the AA site blocks Val/Cys mediated PKM2 inhibition. In complete contrast, PKM2 N75L was inhibited by Val/Cys resulting in a decrease in its catalytic efficiency (Fig 1D, S1 Table). The decrease in the activity of the PKM2 N75L variant in the presence of the AA inhibitors is due to the weakening of PEP binding affinity (*K*_*d*_ ~ 4 μM (-AA) vs. ~ 24 μM (+Val)/ ~ 150 μM (+Cys)) (Fig 2F inset, S2 Table). The *k*_*cat*_*/K*_*m*_ of PKM2 N75L remained unchanged in the presence of Asn/Asp, which is contrary to their effects on wtPKM2 [17], or PKM2 N70D (Fig 1C, 1D, S2A Fig, S1 Table). Likewise, the affinity of PKM2 N75L for PEP (*K*_*d*_ ~3–5 μM) remains unaltered in the presence of AA activators (Fig 2F, S2 Table). The decrease in activity and PEP binding affinity of PKM2 N75L in the presence of Val/Cys are reflected in changes in its oligomeric state, shifting from a mixture of primarily tetramer and some dimer/monomer equilibrium (-AA) to a mixture consisting of nearly equal proportion of tetramer and dimer/monomer equilibrium in the presence of the AA inhibitors (Fig 4D). As expected, the binding affinity of PKM2 N75L for the activator and inhibitor AAs used in the study were similar to wtPKM2, considering that N75 residue is located in the active site and not involved in AA binding (S3 Table). Also, due to the presence of N75 in the active site of PKM2 and its indirect interaction with oxalate (an enolpyruvate analog [23]), the PKM2 N75L mutant undergoes a 100-fold reduction in catalytic activity compared to wtPKM2 (Fig 1B, S1C Fig, S1 Table). However, the order of magnitude difference between the enzymatic activities of wtPKM2 and PKM2 N75L does not influence the effect of the AA activators and inhibitors on PKM2 N75L activity. This is evident from the PEP binding and gel filtration studies of PKM2 N75L, where the effect of the AAs correlates with the activity. In conclusion, the results of PKM2 N75L with the AAs demonstrate that N75 breaks the flow of transmitting the activating signal from the AA binding pocket to the active site but not the inhibition signal, thus confirming that N75 is involved in activation signal transmission. Given PKM2 N75L is responsive to Val/Cys mediated inhibition, it suggests that alternate routes/residues exist that are involved in transmitting the inhibitory signal.

The crystal structures of wtPKM2 bound to various AAs showed that the side chain of R106 flipped towards the AA binding site and anchors to the carboxylate moiety of the bound AA [15, 17, 22]. Whereas, in the absence of any AA, the side chain of R106 is flipped away from the AA binding site as seen with PKM2-FBP structure (PDB: 1T5A) [5]. As R106 is responsible for binding AAs, we hypothesized that it might also be involved in transmitting the allosteric signal from the AA binding pocket to the active site. To test the hypothesis PKM2 R106A variant was investigated in the present study. Interestingly, the mutation alone causes a slight decrease in its activity compared to wtPKM2 (Fig 1B). The influence of Asn/Asp, or Val/Cys on the activation or inhibition of PKM2 R106A followed the same pattern as wtPKM2 (Fig 1E, S1 Table). The activating and inhibiting AAs were able to increase or decrease the PEP binding affinity of PKM2 R106A, respectively, which contributed to the altered activity observed by the AAs (Figs 1E and 2D, S2 Table). Similar to wtPKM2, gel filtration results of PKM2 R106A show a shift from a tetramer (no AA) to a mixture of tetramer and dimer/monomer equilibrium (in the presence of Val/Cys), which is consistent with the decrease in the activity of the variant in the presence of the inhibitory AAs (Figs 1E and 4C). Although the PKM2 R106A variant exhibits similar activation and inhibition patterns compared to wtPKM2, the binding affinities of the AAs for the variant were much weaker relative to wtPKM2 (Fig 3, S3 Table). Thus, the arginine to alanine mutation at 106 does not affect the AA mediated activation or inhibition, but it simply alters the binding affinity of the AAs.

## Conclusions

In summary, the biochemical studies described here demonstrate that N70 and N75 form S1two ends of a signaling pathway transferring the allosteric signal from the AA binding site to the active site of PKM2. In particular, N70, located in the AA binding site, is involved in transmitting the inhibitory signal, whereas N75, located near the active site, is involved in activation signal transmission. R106 is involved solely in anchoring the AAs in the binding pocket and does not take part in the signal transfer process. The study further raises the possibility of alternative residues and routes involved in signal transmission. Moreover, it is possible that the β1 and β2 strands function in a coordinated manner to control the activation/inhibition activity of PKM2. Thus, future studies can be directed towards additional mutagenesis studies of residues forming the two ends of the β2 strand.

## Supporting information

S1 Fig Structures of PKM2 tetramer, monomer, and active site. (A) Superimposition of the overall structure of PKM2 N70D (PDB: 7L21, yellow) on wtPKM2-FBP (PDB:1T5A, cyan). Dotted lines represent the interface of PKM2 monomers. (B) Monomer of PKM2 N70D (chain A) superimposed on PKM2-Cys (PDB:6NU1, brown) and wtPKM2-FBP. The spheres represent oxalate (blue), Cys (green), and FBP (red) located in the active site, AA- and FBP-binding sites of PKM2 respectively. Oxalate and FBP displayed are from the wtPKM2-FBP structure, and Cys is from the PKM2-Cys structure. The distances from active site to AA binding site, and AA binding site to FBP binding site is shown by dashed arrows. (C) Active site of wtPKM2 (PDB:1T5A, cyan) depicting the interaction between N75, K^+^ (violet), water (red), and Mg^2+^ (green), and oxalate. The H-bond interactions are shown as dashed lines. O, N, and C atoms are in red, blue, and backbone color, respectively.
(TIFF)

S2 Fig Effect of activating (Asn, Asp) and inhibiting (Val, Cys) AAs on the activity of wtPKM2 and PKM2 variants. (A) Asn and Asp increase the *k*_*cat*_*/K*_*m*_ of wtPKM2 by ~8 fold, whereas Val or Cys decreases it by ~4 fold. The ADP, AA, and enzyme concentrations were kept constant at 0.8 mM, 2 mM, and 10 nM, respectively. PEP concentration was varied between 0.15–8 mM. Michaelis-Menten curves of: (B) wtPKM2 (C) PKM2 N70D (D) PKM2 N75L (E) PKM2 R106A. Line colors: No AA (black), +Asn (magenta), +Asp (orange), +Val (green), +Cys (brown).
(TIFF)

S3 Fig Substrate binding studies of wtPKM2, PKM2 N70D, and PKM2 N75L. (A)—(B) ADP and PEP binding studies of wtPKM2 in the presence of Cys (brown) respectively. (C)–(D) ADP binding studies of PKM2 N70D and PKM2 N75L, respectively, in the absence (black) and presence of Asn (magenta), Asp (salmon), Val (green), and Cys (brown). The ADP binding affinity of both the variants remains unchanged in the presence and absence of these AAs. The enzyme and AA concentrations were kept constant at 1.6 μM and 1 mM, respectively. The *K*_*d*_ values are listed in S2 Table.
(TIFF)

S4 Fig Gel filtration chromatogram of wtPKM2 in the absence (black) and presence of Cys (brown). The enzyme and AA concentrations used for all the experiments were 0.1 mg/ml and 10 mM, respectively. The standards (158 kDa and 44 kDa) and theoretical molecular weight of dimer (120 kDa) and monomer (60 kDa) are marked as ticks on the upper axis.
(TIFF)

S5 Fig FBP binding affinity of PKM2 N70D. The *K*_*d*_ of PKM2 N70D for FBP was determined to be 1.9768±0.0002 nM, whereas, for wtPKM2, it is 1 nM ^1^. The enzyme concentration was 1.6 μM.
(TIFF)

S1 Table Kinetic parameters of wtPKM2 and PKM2 variants in the absence and presence of Asn, Asp, Val, and Cys. Related to Fig 1 and S2 Fig. The activity assays were performed with 65 nM PKM2 N70D, 4200 nM PKM2 N75L, and 50 nM PKM2 R106A. The concentration of PEP was varied between 0.025–8 mM and the ADP concentration was kept constant at 0.8 mM for all assays. The AA concentrations were fixed at 2 mM for all experiments, with the exception of PKM2 R106A, where 10 mM Val was used.
(PDF)

S2 Table Dissociation constants (*K*_*d*_) of wtPKM2 and PKM2 variants for substrates ADP and PEP in the presence and absence of AA ligands, Asn, Asp, Val, and Cys. The enzyme concentration used for all experiments was 1.6 μM and all the AA ligand concentrations were 1 mM. Related to Fig 2 and S2 Fig.
(PDF)

S3 Table Determination of binding affinities (*K*_*d*_) of PKM2 variants for various AAs and comparison with wtPKM2. The concentration of each enzyme was kept constant at 1.6 μM. The *K*_*d*_ values are reported in μM. Related to Fig 3.
(PDF)

## References

[1] Ashizawa K, McPhie P, Lin KH, Cheng SY. An in vitro novel mechanism of regulating the activity of pyruvate kinase M2 by thyroid hormone and fructose 1, 6-bisphosphate. Biochemistry. 1991;30(29):7105–11. doi: 10.1021/bi00243a010 1854723

[2] Wu S, Le H. Dual roles of PKM2 in cancer metabolism. Acta biochimica et biophysica Sinica. 2013;45(1):27–35. doi: 10.1093/abbs/gms106 23212076

[3] Munday KA, Giles IG, Poat PC. Review of the Comparative Biochemistry of Pyruvate-Kinase. Comp Biochem Physiol B. 1980;67(3):403–11. 10.1016/0305-0491(76)90315-1 975778

[4] Yang WW, Lu ZM. Pyruvate kinase M2 at a glance. J Cell Sci. 2015;128(9):1655–60. doi: 10.1242/jcs.166629 25770102 PMC4446733

[5] Dombrauckas JD, Santarsiero BD, Mesecar AD. Structural basis for tumor pyruvate kinase M2 allosteric regulation and catalysis. Biochemistry. 2005;44(27):9417–29. doi: 10.1021/bi0474923 15996096

[6] Yan M, Chakravarthy S, Tokuda JM, Pollack L, Bowman GD, Lee YS. Succinyl-5-aminoimidazole-4-carboxamide-1-ribose 5’-Phosphate (SAICAR) Activates Pyruvate Kinase Isoform M2 (PKM2) in Its Dimeric Form. Biochemistry. 2016;55(33):4731–6. doi: 10.1021/acs.biochem.6b00658 27481063 PMC4995138

[7] Keller KE, Tan IS, Lee YS. SAICAR stimulates pyruvate kinase isoform M2 and promotes cancer cell survival in glucose-limited conditions. Science. 2012;338(6110):1069–72. doi: 10.1126/science.1224409 23086999 PMC3527123

[8] Lv L, Xu YP, Zhao D, Li FL, Wang W, Sasaki N, et al. Mitogenic and oncogenic stimulation of K433 acetylation promotes PKM2 protein kinase activity and nuclear localization. Mol Cell. 2013;52(3):340–52. doi: 10.1016/j.molcel.2013.09.004 24120661 PMC4183148

[9] Hitosugi T, Kang S, Vander Heiden MG, Chung TW, Elf S, Lythgoe K, et al. Tyrosine phosphorylation inhibits PKM2 to promote the Warburg effect and tumor growth. Sci Signal. 2009(97): doi: 10.1126/scisignal.2000431 19920251 PMC2812789

[10] Yang W, Zheng Y, Xia Y, Ji H, Chen X, Guo F, et al. ERK1/2-dependent phosphorylation and nuclear translocation of PKM2 promotes the Warburg effect. Nat Cell Biol. 2012;14(12):1295–304. doi: 10.1038/ncb2629 23178880 PMC3511602

[11] Yang W, Xia Y, Ji H, Zheng Y, Liang J, Huang W, et al. Nuclear PKM2 regulates beta-catenin transactivation upon EGFR activation. Nature. 2011;480(7375):118–22. 22056988 10.1038/nature10598 PMC3235705

[12] Luo W, Hu H, Chang R, Zhong J, Knabel M, O’Meally R, et al. Pyruvate kinase M2 is a PHD3-stimulated coactivator for hypoxia-inducible factor 1. Cell. 2011;145(5):732–44. doi: 10.1016/j.cell.2011.03.054 21620138 PMC3130564

[13] Anastasiou D, Yu Y, Israelsen WJ, Jiang JK, Boxer MB, Hong BS, et al. Pyruvate kinase M2 activators promote tetramer formation and suppress tumorigenesis. Nat Chem Biol. 2012;8(10):839–47. doi: 10.1038/nchembio.1060 22922757 PMC3711671

[14] Yacovan A, Ozeri R, Kehat T, Mirilashvili S, Sherman D, Aizikovich A, et al. 1-(sulfonyl)-5-(arylsulfonyl)indoline as activators of the tumor cell specific M2 isoform of pyruvate kinase. Bioorg Med Chem Lett. 2012;22(20):6460–8. doi: 10.1016/j.bmcl.2012.08.054 22963766

[15] Srivastava D, Nandi S, Dey M. Mechanistic and Structural Insights into Cysteine-Mediated Inhibition of Pyruvate Kinase Muscle Isoform 2. Biochemistry. 2019;58:3669–82. doi: 10.1021/acs.biochem.9b00349 31386812

[16] Chaneton B, Hillmann P, Zheng L, Martin ACL, Maddocks ODK, Chokkathukalam A, et al. Serine is a natural ligand and allosteric activator of pyruvate kinase M2. Nature. 2012;491(7424):458–+. doi: 10.1038/nature11540 23064226 PMC3894725

[17] Nandi S, Dey M. Biochemical and structural insights into how amino acids regulate pyruvate kinase muscle isoform 2. J Biol Chem. 2020;295(16):5390–403. doi: 10.1074/jbc.RA120.013030 32144209 PMC7170521

[18] Damaghi M, Wojtkowiak JW, Gillies RJ. pH sensing and regulation in cancer. Front Physiol. 2013;4:370. doi: 10.3389/fphys.2013.00370 24381558 PMC3865727

[19] Yang W, Xia Y, Hawke D, Li X, Liang J, Xing D, et al. PKM2 phosphorylates histone H3 and promotes gene transcription and tumorigenesis. Cell. 2012;150(4):685–96. doi: 10.1016/j.cell.2012.07.018 22901803 PMC3431020

[20] Nandi S, Razzaghi M, Srivastava D, Dey M. Structural basis for allosteric regulation of pyruvate kinase M2 by phosphorylation and acetylation. J Biol Chem. 2020. doi: 10.1074/jbc.RA120.015800 33453989 PMC7762928

[21] Nakatsu D, Horiuchi Y, Kano F, Noguchi Y, Sugawara T, Takamoto I, et al. L-cysteine reversibly inhibits glucose-induced biphasic insulin secretion and ATP production by inactivating PKM2. Proc Natl Acad Sci USA. 2015;112(10):E1067–76. doi: 10.1073/pnas.1417197112 25713368 PMC4364213

[22] Yuan M, McNae IW, Chen YY, Blackburn EA, Wear MA, Michels PAM, et al. An allostatic mechanism for M2 pyruvate kinase as an amino-acid sensor. Biochem J 2018;475:1821–37. doi: 10.1042/BCJ20180171 29748232 PMC5980995

[23] Seeholzer SH, Jaworowski A, Rose IA. Enolpyruvate: chemical determination as a pyruvate kinase intermediate. Biochemistry. 1991;30(3):727–32. doi: 10.1021/bi00217a022 1988060

[24] Brummett AE, Schnicker NJ, Crider A, Todd JD, Dey M. Biochemical, Kinetic, and Spectroscopic Characterization of Ruegeria pomeroyi DddW—A Mononuclear Iron-Dependent DMSP Lyase. PLoS One. 2015;10(5):e0127288. doi: 10.1371/journal.pone.0127288 25993446 PMC4437653

[25] Xds Kabsch W. Acta Crystallogr D. 2010;66(Pt 2):125–32. 20124692 10.1107/S0907444909047337 PMC2815665

[26] Winn MD, Ballard CC, Cowtan KD, Dodson EJ, Emsley P, Evans PR, et al. Overview of the CCP4 suite and current developments. Acta Crystallogr D. 2011;67(Pt 4):235–42. doi: 10.1107/S0907444910045749 21460441 PMC3069738

[27] McCoy AJ, Grosse-Kunstleve RW, Adams PD, Winn MD, Storoni LC, Read RJ. Phaser crystallographic software. J Appl Crystallogr. 2007;40(Pt 4):658–74. doi: 10.1107/S0021889807021206 19461840 PMC2483472

[28] Adams PD, Afonine PV, Bunkoczi G, Chen VB, Davis IW, Echols N, et al. PHENIX: a comprehensive Python-based system for macromolecular structure solution. Acta Crystallogr D. 2010;66(Pt 2):213–21. 20124702 10.1107/S0907444909052925 PMC2815670

[29] Emsley P, Lohkamp B, Scott WG, Cowtan K. Features and development of Coot. Acta Crystallogr D. 2010;66(Pt 4):486–501. doi: 10.1107/S0907444910007493 20383002 PMC2852313

[30] Berman HM, Kleywegt GJ, Nakamura H, Markley JL. The future of the Protein Data Bank. Biopolymers. 2013;99(3):218–22. doi: 10.1002/bip.22132 23023942 PMC3684242

[31] Delano W. PyMOL: An open-source molecular graphics tool. CCP4 Newsletter on Protein Crystallography. 2002;40:82–92.

[32] Kalaiarasan P, Kumar B, Chopra R, Gupta V, Subbarao N, Bamezai RN. In silico screening, genotyping, molecular dynamics simulation and activity studies of SNPs in pyruvate kinase M2. PLoS One. 2015: doi: 10.1371/journal.pone.0120469 25768091 PMC4359060

