# Supplementary figures and images for: Identification of residues involved in allosteric signal transmission from amino acid binding site of pyruvate kinase muscle isoform 2
Source: PLoS One. 2023 Mar 10;18(3):e0282508. doi: 10.1371/journal.pone.0282508 (PMC10004559; doi:10.1371/journal.pone.0282508)

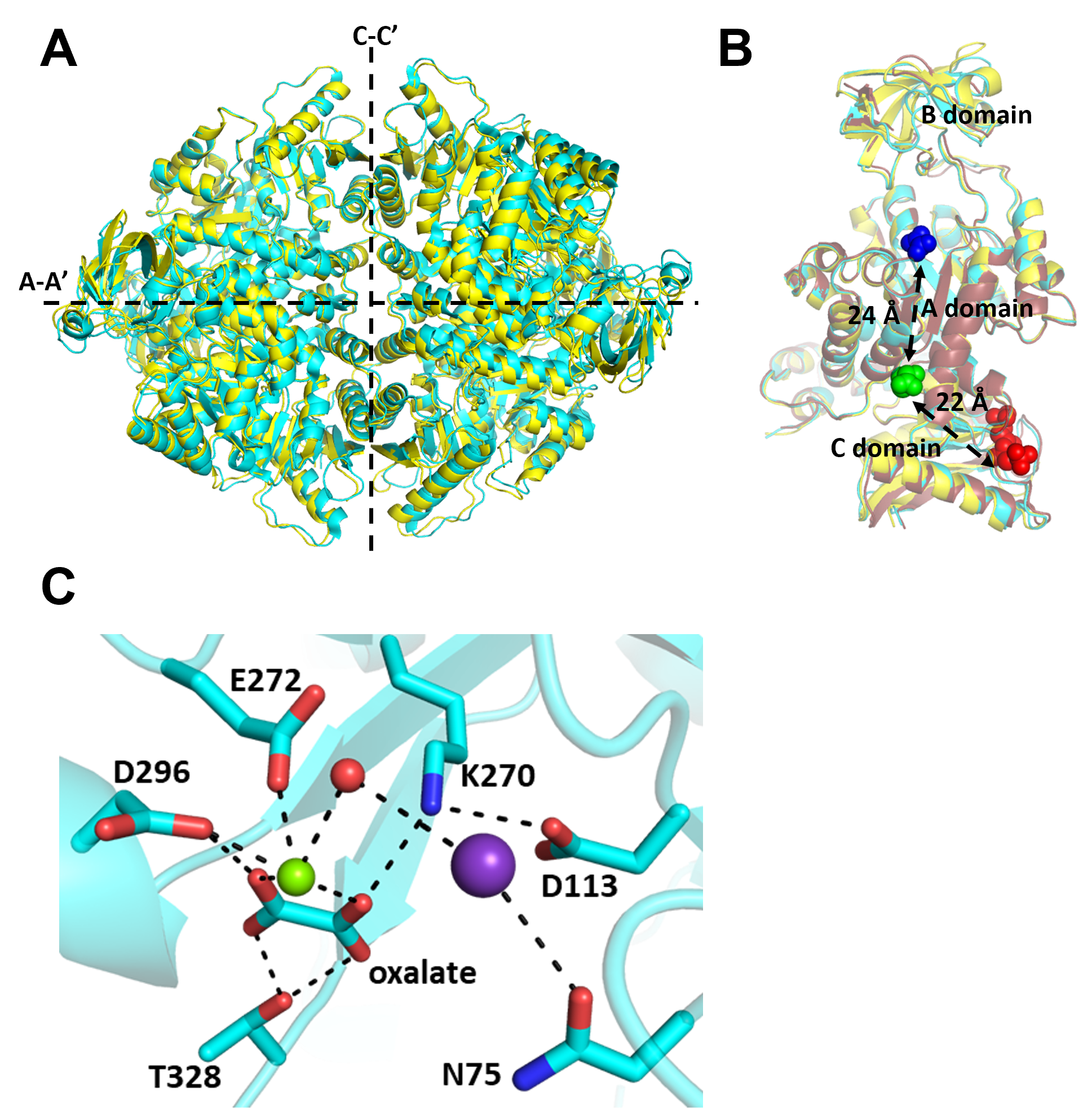

Supplement: S1 Fig — (A) Superimposition of the overall structure of PKM2 N70D (PDB: 7L21, yellow) on wtPKM2-FBP (PDB:1T5A, cyan). Dotted lines represent the interface of PKM2 monomers. (B) Monomer of PKM2 N70D (chain A) superimposed on PKM2-Cys (PDB:6NU1, brown) and wtPKM2-FBP. The spheres represent oxalate (blue), Cys (green), and FBP (red) located in the active site, AA- and FBP-binding sites of PKM2 respectively. Oxalate and FBP displayed are from the wtPKM2-FBP structure, and Cys is from the PKM2-Cys structure. The distances from active site to AA binding site, and AA binding site to FBP binding site is shown by dashed arrows. (C) Active site of wtPKM2 (PDB:1T5A, cyan) depicting the interaction between N75, K+ (violet), water (red), and Mg2+ (green), and oxalate. The H-bond interactions are shown as dashed lines. O, N, and C atoms are in red, blue, and backbone color, respectively. (TIFF) [file pone.0282508.s001.tiff]

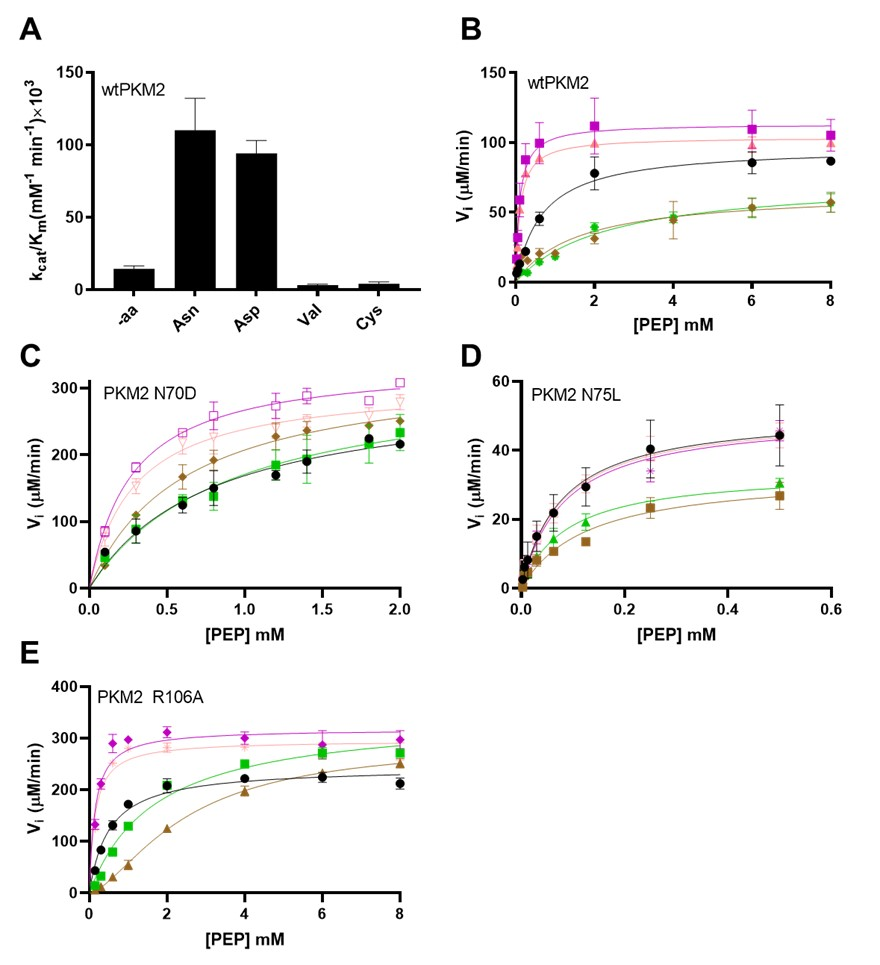

Supplement: S2 Fig — (A) Asn and Asp increase the kcat/Km of wtPKM2 by ~8 fold, whereas Val or Cys decreases it by ~4 fold. The ADP, AA, and enzyme concentrations were kept constant at 0.8 mM, 2 mM, and 10 nM, respectively. PEP concentration was varied between 0.15–8 mM. Michaelis-Menten curves of: (B) wtPKM2 (C) PKM2 N70D (D) PKM2 N75L (E) PKM2 R106A. Line colors: No AA (black), +Asn (magenta), +Asp (orange), +Val (green), +Cys (brown). (TIFF) [file pone.0282508.s002.tiff]

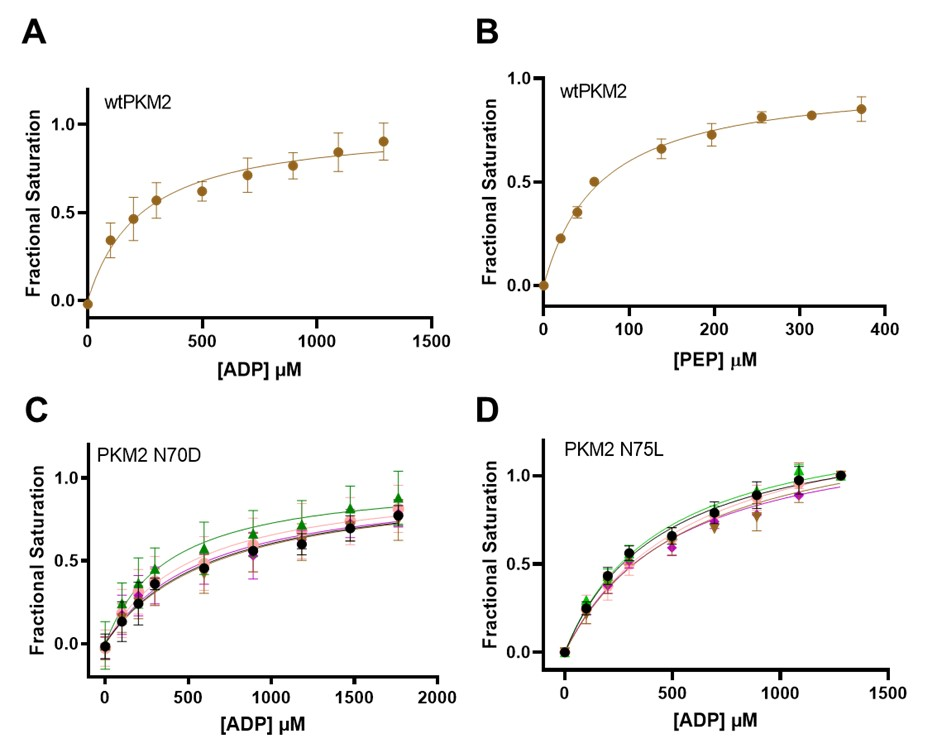

Supplement: S3 Fig — (A)—(B) ADP and PEP binding studies of wtPKM2 in the presence of Cys (brown) respectively. (C)–(D) ADP binding studies of PKM2 N70D and PKM2 N75L, respectively, in the absence (black) and presence of Asn (magenta), Asp (salmon), Val (green), and Cys (brown). The ADP binding affinity of both the variants remains unchanged in the presence and absence of these AAs. The enzyme and AA concentrations were kept constant at 1.6 μM and 1 mM, respectively. The Kd values are listed in S2 Table. (TIFF) [file pone.0282508.s003.tiff]

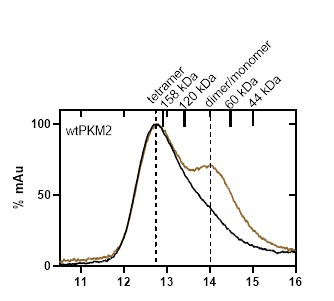

Supplement: S4 Fig — The enzyme and AA concentrations used for all the experiments were 0.1 mg/ml and 10 mM, respectively. The standards (158 kDa and 44 kDa) and theoretical molecular weight of dimer (120 kDa) and monomer (60 kDa) are marked as ticks on the upper axis. (TIFF) [file pone.0282508.s004.tiff]

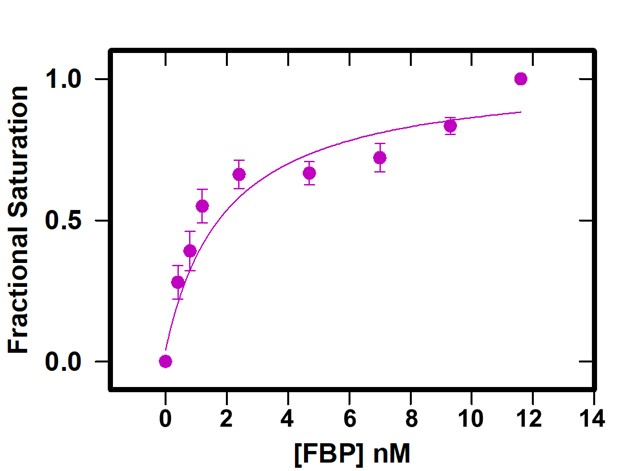

Supplement: S5 Fig — The Kd of PKM2 N70D for FBP was determined to be 1.9768±0.0002 nM, whereas, for wtPKM2, it is 1 nM 1. The enzyme concentration was 1.6 μM. (TIFF) [file pone.0282508.s005.tiff]
