# Supplementary material for: Identification of residues involved in allosteric signal transmission from amino acid binding site of pyruvate kinase muscle isoform 2
Source: PLoS One. 2023 Mar 10;18(3):e0282508. doi: 10.1371/journal.pone.0282508 (PMC10004559; doi:10.1371/journal.pone.0282508)
Supplement: S1 Table — Related to Fig 1 and S2 Fig. The activity assays were performed with 65 nM PKM2 N70D, 4200 nM PKM2 N75L, and 50 nM PKM2 R106A. The concentration of PEP was varied between 0.025–8 mM and the ADP concentration was kept constant at 0.8 mM for all assays. The AA concentrations were fixed at 2 mM for all experiments, with the exception of PKM2 R106A, where 10 mM Val was used. (PDF) [file pone.0282508.s006.pdf]

wtPKM2 and PKM2 variants	AAs	k_{cat} ×10^{3} (min^{-1})	K_{m}^{PEP} (mM)	k_{cat} /K_{m}^{PEP} ×10^{3} (mM^{-1} min^{-1})	V_{max} (μM/min)
wtPKM2	--	9.7±0.4	0.7±0.1	13.8±2	97±4
	Val	7.4±0.5	2.3±0.4	3.2±0.6	74±5
	Cys	6.5±0.7	1.6±0.5	4.1±1.3	65±7
	Asn	11.3±0.4	0.10±0.02	113±23	113±4
	Asp	10.4±0.2	0.11±0.01	94.5±8.8	104±2
PKM2 N70D	--	4.6±0.4	0.78±0.16	5.9±1.3	301±24
	Val	4.9±0.6	0.89±0.24	5.5±1.6	324±37
	Cys	5.1±0.2	0.61±0.07	8.4±1.0	334±15
	Asn	5.2±0.3	0.27±0.03	19.2±2.4	340±8
	Asp	4.7±0.1	0.29±0.04	16.2±2.3	307±10
PKM2 N75L	--	0.012±0.001	0.08±0.02	0.15±0.04	51±4
	Val	0.008±0.0	0.09±0.01	0.08±0.01	34±2
	Cys	0.008±0.0	0.13±0.03	0.06±0.01	33±3
	Asn	0.012±0.0	0.08±0.01	0.15±0.02	50±2
	Asp	0.012±0.0	0.08±0.01	0.15±0.02	51±3
PKM2 R106A	--	4.9±0.1	0.51±0.04	9.6±0.8	244±5
	Val	6.9±0.2	1.7±0.17	4.0±0.4	346±12
	Cys	5.8±0.2	4.0±0.2	1.4±0.1	291±9
	Asn	6.3±0.2	0.14±0.02	45±6	318±8
	Asp	5.9±0.1	0.15±0.02	39±5	295±6
