# Supplementary material for: Identification of residues involved in allosteric signal transmission from amino acid binding site of pyruvate kinase muscle isoform 2
Source: PLoS One. 2023 Mar 10;18(3):e0282508. doi: 10.1371/journal.pone.0282508 (PMC10004559; doi:10.1371/journal.pone.0282508)
Supplement: S2 Table — The enzyme concentration used for all experiments was 1.6 μM and all the AA ligand concentrations were 1 mM. Related to Fig 2 and S2 Fig. (PDF) [file pone.0282508.s007.pdf]

wtPKM2 and PKM2 variants	Ligand	K_d (µM), ADP as titrant	K_d (µM), PEP as titrant
wtPKM2	--	237±54 (2)	10±2 (2)
	Val	363±57 (2)	81±21 (2)
	Cys	237±54	66.7±5.7
	Asn	345±33 (2)	0.27±0.0 (2)
	Asp	585±188 (2)	0.29±0.01(2)
PKM2 N70D	--	644±154	40.3±5.1
	Val	382±125	21.7±1.7
	Cys	670±212	40.6±7.1
	Asn	600±185	2.2±0.8
	Asp	525±200	2.1±0.7
PKM2 N75L	--	449±48	3.5±0.8
	Val	448±41	23.6±2.8
	Cys	568±99	152.2±20.8
	Asn	521±59	4.4±0.5
	Asp	576±62	5.0±0.6
PKM2 R106A	--	257±24	9.5±1.1
	Val	1211±165	26.5±7.3
	Cys	2883±415	100.3±29
	Asn	208±24	1.0±0.0
	Asp	220±21	1.0±0.0
