# Supplementary material for: Identification of residues involved in allosteric signal transmission from amino acid binding site of pyruvate kinase muscle isoform 2
Source: PLoS One. 2023 Mar 10;18(3):e0282508. doi: 10.1371/journal.pone.0282508 (PMC10004559; doi:10.1371/journal.pone.0282508)
Supplement: S3 Table — The concentration of each enzyme was kept constant at 1.6 μM. The Kd values are reported in μM. Related to Fig 3. (PDF) [file pone.0282508.s008.pdf]

**Enzyme**	Asn	Asp	Val	Cys
wtPKM2	11±2 (1)	35±7 (1)	757±120 (1)	11±3 (1)
PKM2 N70D	12±2	38±10	590±119	6.1±1.1
PKM2 N75L	14±2	28±6	611±115	9±2
PKM2 R106A	42±10	73±21	1626±276	36±5

1. Nandi S, Dey M. Biochemical and structural insights into how amino acids regulate pyruvate kinase muscle isoform 2. J Biol Chem. 2020;295(16):5390-403.
